# Coordination of drought‐adaptive root growth plasticity through auxin homeostasis by the ZmSPL12–*ZmYUC5* module in maize

**DOI:** 10.1111/nph.71592

**Published:** 2026-09-22

**Authors:** Hua Xu, Lei Ma, Qingyun Zhang, Zhigang Zheng, Jiajing Li, Sen Yang, Yanfeng Zhao, Lian Jin, Binbin Zhao, Zhijing Bai, Jiyuan Cao, Youpeng Tao, Junjie Zou, Baobao Wang, Shujing Cheng, Jinfang Chu, Jiafan Liu, Haiyang Wang, Miaoyun Xu

**Affiliations:** ^1^ Biotechnology Research Institute Chinese Academy of Agricultural Sciences Beijing 100081 China; ^2^ National Nanfan Research Institute CAAS Sanya 572025 China; ^3^ Yazhouwan National Laboratory Sanya 572025 China; ^4^ The Shennong Laboratory Xinxiang 453500 China; ^5^ National Engineering Laboratory of Crop Stress Resistance Breeding, College of Life Sciences Anhui Agricultural University Hefei 230036 China; ^6^ State Key Laboratory of Seed Innovation, National Center for Plant Gene Research (Beijing) Institute of Genetics and Developmental Biology, Chinese Academy of Sciences Beijing 100101 China; ^7^ University of Chinese Academy of Science Beijing 100049 China

**Keywords:** auxin homeostasis, drought adaptation, maize, root plasticity, yield stability, ZmSPL12

## Abstract

- Climate change‐induced drought increasingly threatens global maize production. Drought‐adaptive root plasticity in maize depends on auxin signaling, yet how auxin homeostasis is dynamically tuned in response to water deficit remains unclear. This study investigates the transcription factor SQUAMOSA PROMOTER‐BINDING PROTEIN‐LIKE 12 (ZmSPL12) as a context‐dependent modulator of root growth plasticity under drought stress (DS).
- We combined phenotypic phenotyping of transgenic overexpression and knockout maize lines, molecular biochemical assays, DAP‐seq, hormone quantification, and field drought trials of inbred lines and commercial hybrids.
- *ZmSPL12* overexpression produces shorter roots under well‐watered conditions yet sustains root elongation under DS, conferring enhanced drought adaptation. ZmSPL12 directly binds to the promoter of the auxin biosynthesis gene *ZmYUC5* and suppresses its expression, establishing a buffered basal auxin state in root tips, preventing drought‐triggered auxin overaccumulation that inhibits meristem activity. Field trials show that *ZmSPL12* overexpression enhances drought resilience in inbred lines and improves grain yield stability in commercial hybrids under water‐limited conditions.
- The ZmSPL12–*ZmYUC5* module acts as a molecular rheostat that fine‐tunes root‐tip auxin homeostasis to coordinate drought‐adaptive root architectural plasticity in maize. This work reveals a conditional‐plasticity regulatory mechanism and offers a valuable genetic resource for breeding climate‐resilient crops.

## Graphical abstract

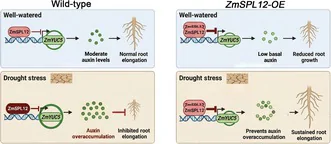

## Introduction

Drought is the most damaging environmental stressor causing a global average annual yield loss of 19.18% in maize (*Zea mays* L.; Guo *et al*., 2016), the world's highest‐yielding cereal crop (Ahuja *et al*., 2010; McElwain, 2018; Sun & Fernie, 2024). Climate change is intensifying the frequency and severity of drought events (Tang *et al*., 2025), while agricultural water demand is projected to double by 2050 amid a sharp decline in freshwater resources (Gupta *et al*., 2020). Breeding drought‐resistant maize cultivars is essential for sustainable agriculture. This requires a comprehensive dissection of the genetic basis of drought resistance and identifying key genes that enhance resilience without limiting growth or yield.

Drought tolerance involves complex quantitative traits governed by morphological adaptations – such as root growth and architecture, and leaf hydraulics – as well as physiological processes (Gupta *et al*., 2020; Rasheed *et al*., 2023). The root system acts as the primary sensor of soil water deficit and plays a central role in coordinating adaptive responses (Bengough *et al*., 2006; Meister *et al*., 2014; Rogers & Benfey, 2015; Hochholdinger, 2016; Van der Bom *et al*., 2020). Under drought, roots elongate and proliferate toward water‐rich zones to improve water uptake and sustain plant development (Comas *et al*., 2013; Feng *et al*., 2022). In maize, the root system comprises embryonic primary and seminal roots, postembryonic shoot‐borne crown roots, and brace roots (Hochholdinger, 2009; Atkinson *et al*., 2014). The primary and seminal roots support early seedling vigor, whereas shoot‐borne roots form the structural backbone of the mature root system (Hochholdinger, 2009). All root types have the potential to form lateral roots from dividing pericycle cells post embryonically (Hochholdinger *et al*., 2004), thereby enhancing nutrient and water absorption by expanding the root surface area (P. Yu *et al*., 2019). Studies have confirmed that a reduced lateral root branching density enhances drought tolerance in maize by facilitating deeper rooting and water acquisition (Hochholdinger, 2009; Thorup‐Kristensen *et al*., 2020; Voothuluru *et al*., 2024). This aligns with the ‘steep, deep, and cheap’ maize root ideotype proposed by Lynch to enhance drought tolerance (Lynch, 2013; Van Oosterom *et al*., 2016). This architectural strategy has been validated in several crops including maize (Zhu *et al*., 2010; Chimungu *et al*., 2014a,b). It is exploited in the agronomic practice of ‘seedling drought‐hardening’ to promote morphological adaptations like an increased root‐to‐shoot ratio and deeper root systems, thereby enhancing water acquisition and drought resilience (Huang *et al*., 2013).

Root tissues originate from asymmetric divisions of stem cells adjacent to the quiescent center, together forming the stem cell niche. The stem cells' progeny proliferates in the meristematic zone before ceasing division at the transition boundary (Benfey & Scheres, 2000; Dello Ioio *et al*., 2007). Here, cells begin to elongate and differentiate, progressing through subsequent differentiation stages until maturation. The coordinated activity of these zones maintains the position of the transition zone (TZ), which is essential for stabilizing meristem size and ensuring continuous root growth (Bennett, 2020). The TZ is positioned by an auxin minimum, which is shaped by the antagonistic interaction of auxin and cytokinin to trigger the shift from proliferation to differentiation and control root growth (Dello Ioio *et al*., 2008; Di Mambro *et al*., 2017). Previous studies have demonstrated that auxin accumulates in root cortical tissues during drought stress (DS; Rowe *et al*., 2016; Abualia, 2023), as evidenced by the induction of auxin biosynthesis genes like *GH3.13* and *YUCCA* in drought‐resistant plants (Lee *et al*., 2012; Kim *et al*., 2013; Shi *et al*., 2014). Consistent with this, rice mutants of *OsCOW1*, which encodes a YUCCA family member, exhibit a water‐deficient phenotype with impaired water uptake (Woo *et al*., 2007). In maize, auxin promotes root growth toward deeper soil layers, and abscisic acid (ABA) regulates the expression or activity of auxin transport proteins (such as PIN proteins), affecting the distribution of auxin in root tips and guiding root growth toward deeper soil layers with richer water content (Yang *et al*., 2023). *ZmARF1* affects drought resistance by regulating root development (Liu *et al*., 2024). Overexpression of *ZmPIN1a* can enhance maize root system and improve drought resistance (Li *et al*., 2018). Under DS, elevated auxin levels promote both cell proliferation (Dello Ioio *et al*., 2008; Di Mambro *et al*., 2017) and elongation in the root tip (Uga *et al*., 2013), yet how plants maintain optimal auxin concentration in the TZ – where low auxin triggers the shift from division to differentiation – remains unclear. Furthermore, the molecular mechanisms connecting drought perception to auxin‐mediated root plasticity are still poorly understood.

The family of SQUAMOSA PROMOTER‐BINDING PROTEIN‐LIKE (SPL) transcription factors plays vital roles in regulating plant development and stress responses in maize (Wei *et al*., 2018). We previously reported that overexpressing *ZmSPL12* reduces plant height and confers stronger lodging resistance than its nontransgenic controls, thereby facilitating high‐density planting (Zhao *et al*., 2022). Additionally, overexpression of *ZmSPL12* suppresses maize root growth under normal, well‐watered (WW) conditions, but this effect was substantially attenuated under soil compaction stress, resulting in enhanced root penetration ability and elongation, consequently improving compaction resistance (Xu *et al*., 2024). In this study, we report that *ZmSPL12* also acts as a central regulator of drought‐induced root plasticity in maize by directly repressing the expression of the auxin biosynthesis gene *ZmYUC5*, thereby filling in an important gap connecting DS and auxin responses in modulating maize root development and providing a novel genetic target for breeding drought‐resilient maize varieties.

## Materials and Methods

### Plant materials and growth conditions

The overexpression transgenic maize plants and mutants were derived from the C01 inbred line, which was constructed in our laboratory in the early stage (Zhao *et al*., 2022). Synonymous substitutions at the *ZmSPL12* miR156 binding site blocked microRNA‐mediated cleavage for overexpression construction. Ten independent transformants were generated, and two high‐expression lines were screened by reverse transcription‐quantitative polymerase chain reaction (RT‐qPCR). Nontransgenic segregants from selfed progeny served as matched wild‐type (WT) controls for overexpression materials. A separate CRISPR‐Cas9 vector targeting the *ZmSPL12* coding sequence was transformed into C01. Ten editing events were produced, and homozygous mutants were confirmed by PCR and sequencing. Unedited segregants from mutant selfing acted as exclusive WT controls for knockout (KO) lines. The *ZmSPL12* overexpression lines *ZmSPL12‐*OE1 and *ZmSPL12‐*OE2, KO mutants *Zmspl12‐1* and *Zmspl12‐2*, and their matched WT were used for the drought‐resistance tests in the laboratory and field trials.

We developed improved Jing 724 and Jing 92 inbred lines carrying the *ZmSPL12*‐OE transgene through a systematic breeding approach. The transgenic *ZmSPL12*‐OE line was initially crossed with elite maize inbred lines Jing92 and Jing724, followed by five generations of recurrent backcrossing using Jing92/Jing724 as the recurrent parent. From BC_1_F_1_ to BC_5_F_2_ generations, we consistently selected basta‐resistant progeny. Background selection analysis demonstrated > 94% recurrent parent genome recovery in the resulting fixed lines. For field evaluation, we utilized Jingke968, a commercial maize hybrid developed by the Beijing Academy of Agriculture and Forestry Sciences. This hybrid derives from a cross between the female parent Jing724 (X‐group germplasm) and the male parent Jing92 (Huangzaosi group germplasm). In our study, we generated improved hybrid varieties by crossing the enhanced inbred lines. All genotypes were subjected to drought resistance evaluation during reproductive maturity under field conditions. For *in situ* hybridization experiments, we used tissues collected from the standard maize inbred line B73 as a reference material.

### Drought‐resistance tests

For the drought‐resistance tests in the laboratory, seeds for all genotypes were sown in soil (Professional soil: Shandong Lvzhu Agricultural Science and Technology Development Co. Ltd) in pots (10 cm × 8 cm), with a soil water content of *c*. 35% (water content (%) = [(wet soil mass – dry soil mass)/(dry soil mass)] × 100%). All plants were divided into an experimental group and a control group. The control group was irrigated with water every 3 d. The experimental group was irrigated once with 200 ml of water per pot and then stopped watering until a drought‐resistant phenotype appeared. All plants were grown in the glasshouse under a 12 h : 12 h, light : dark photoperiod at 28°C : 25°C and 50% relative humidity. The phenotype appeared after 15 d of plant growth. The plants were photographed when the phenotype was observed for 3 d. After 1 wk of a stable visible phenotype in response to water limitation, the plants were rewatered at 300 ml per pot, and the number of surviving plants was counted 3 d later. The average survival rate was calculated for three replicates, each consisting of at least 15 plants per genotype (*n* ≥ 15).

### Field drought resistance evaluation of *ZmSPL12* overexpression and KO lines

The plants were grown at the Experimental Base of the Biotechnology Research Institute, Wanzhuang International Agricultural High‐tech Industrial Park, Chinese Academy of Agricultural Sciences, Wanzhuang Town, Guangyang District, Langfang City, Hebei Province, China (39°35′28″N, 116°35′53″E). To investigate whether *ZmSPL12* overexpression contributes to yield and root elongation under severe drought conditions, different transgenic lines and their respective control lines were sown in a rain‐proof shed. At least three replicates for each material were randomly sown in rectangular areas of 8.5 m in length and 3.4 m in width across four grids. Replicates were sown in nine rows with a row spacing of *c*. 45 cm. After seed germination and seedling establishment, 15 plants were retained per row, with a spacing between plants of 25 cm. After maturity, all plants in a single plot were harvested together, and the yields under WW and severe DS conditions were measured as weight of ear. The WW condition is defined as a VWC of 20–30% at a depth of 10 cm (Marchin *et al*., 2019), which is maintained until harvest. DS was applied by different watering times, including WW conditions, until the plants reached the V9 stage, followed by water control of the experimental group until harvest. The soil moisture analyzer (LANENDE: LD‐SW) measures the VWC under DS conditions. The height and anthesis–silking interval (ASI) of all plants grown in the two environments were investigated at 60 d after sowing. Plant height was measured from the basal part of the plant to the top of the uppermost tassel. All inbred and improved inbred lines were used for this drought‐resistance test.

Field drought resistance experiments on improved inbred lines and hybrid varieties: Drought‐resistance test in the field was conducted at the Experimental Base of Grain Crop Research Institute, Xinjiang Academy of Agricultural Sciences, Anning District, Urumqi City, China (43°56′32″N, 87°46′45″E). WW conditions were maintained until harvest. The DS treatment was applied from the V9 stage until harvest as half of the irrigation provided for the WW conditions. Yield‐related data were measured as above. All improved inbred lines and hybrids were grown for this test.

Osmotic stress experiment during seedling stage: The experimental materials were rolled into kraft paper for pure water culture, with pure water changed every 2 d. All plants were grown in the glasshouse under a 12 h : 12 h, light : dark photoperiod at 28°C and 40% relative humidity. After culturing in water for 1 wk, half of the materials were treated with 15% PEG6000 for culture, and the other half continued to be cultured in pure water. The seedlings were photographed after 3, 5, and 7 d of growth, and the primary root length was measured with a ruler. Root tips cultured for 5 d were collected for sectioning, quantitative assays, and hormone determination.

### Root phenotyping and data analysis

To observe the changes in root length at the seedling stage in soil, the root systems of seedlings exposed to DS for 20 d were washed with water to remove roots with poor development or damaged roots and remove soil particles. Photographs were taken of the root systems with a Canon 60D camera. Primary root length was measured with a ruler.

To observe the crown root systems in the field, the root systems were accessed by *in situ* excavation. A hoe was first used to excavate the approximate range of the root system. A shovel was then used for further detailed excavation of the root system. Subsequently, a soft brush was employed to gently remove soil particles adhering to the root surface. The crown root length was measured vertically to the ground using a tape measure, with the measurement spanning from the root tip to the soil surface.

### Cell number and length measurement

After root tip sectioning, samples were stained with 0.5% toluidine blue and scanned using a Pannoramic digital slide scanner. Digital section images were viewed with Caseviewer software, in which cell morphology and size were clearly resolved under 40× magnification. This software was further applied to quantify cell number and cell length.

### Quantification of endogenous IAA levels

Approximately 30 mg (fresh weight) of plant tissue was homogenized under liquid nitrogen, weighed and extracted for 24 h with methanol and ^2^H_2_‐IAA. Endogenous IAA was purified and measured as previously described with the following modifications in detection conditions (Fu *et al*., 2012; Chu *et al*., 2017). LC‐MS/MS analysis was performed on a UPLC system (Waters corporation, Milford, MA, USA) coupled to the 5500 Qtrap system (AB Sciex, LLC, Framingham, MA, USA). LC separation used a BEH C18 column (1.7 μm, 100 × 2.1 mm; Waters corporation) with mobile phase A, 0.1% (v/v) formic acid in water, and B, acetonitrile. The gradient was as follows: 0–0.5 min, 5% B, 0.5–14 min, 5% B to 25% B; 14–16 min, 25% B to 100% B. IAA was detected in multiple reaction monitoring mode with transition. The transitions for IAA and [^2^H_2_]‐IAA are 174.1 > 130.1 and 176.1 > 132.1.

### Construction of *ZmYUC5* CRISPR‐Cas9 Vector

The CDS sequence of *ZmYUC5* was cloned and aligned with the maize genome data (https://www.maizegdb.org/). All exons on the genome sequences of these genes were marked as target selection regions. Using the reference target sequence 5 ‘G‐ (N) 20‐NGG 3’ (where N represents any one of the four bases) as a template, use Snapgene viewer software to label candidate target sequences. All candidate target sequences were uploaded to the online Blast analysis tool (https://ensembl.gramene.org), after which specific sequences were selected. Construct *ZmU6‐1/ZmU6‐2: sgRNA* expression cassette and *pCPB Ubi: hspCas9‐ZmYUC5* recombinant plasmid and perform genetic transformation after sequencing validation of the recombinant plasmid (this work was carried out by the Life Science Technology Center of China Seed Group Co. Ltd, Wuhan).

### RNA extraction and RT‐qPCR assays

To analyze the expression of *ZmSPL12* in response to osmotic stress, samples were collected from WT seedlings WT^OE1^ grown in water at 28°C for 2 wk and transferred to 20% (w/v) PEG‐6000. Samples were collected at 0, 1, 2, 3, 4, 5, 6, 9, 12, and 24 h following transfer. Total RNA was extracted with an RNA extraction kit (RC411‐01) provided by Vazyme Biotech Co. Ltd, Nanjing, Jiangsu, China. The quantity and quality of RNA were assessed by measuring the optical density (OD) at 260 nm and 280 nm by a Nanodrop‐2000 Ultraviolet Spectrophotometer (Thermo Fisher Scientific, Waltham, MA, USA). RTqPCR and reverse transcription polymerase chain reaction (RT‐PCR), first‐strand cDNA was synthesized using a QuantiTect Reverse Transcription synthesis kit (R222‐01) provided by Vazyme Biotech Co. Ltd. Gene expression analysis was performed by qRT‐PCR using *ZmTub5* as an internal control. qPCR reactions used SYBR Green I (Tsingke Biotechnology Co. Ltd 2 × T5 Fast qPCR Mix. TSE202) and gene‐specific primers. Relative expression levels were calculated via the 2^−ΔΔCt^ method, with *ZmTub5* normalization and WW WT plants as the control.

To analyze the expression of *ZmYUC5* and *ZmSPL12* in the root tips of *ZmSPL12*‐OE, *ZmSPL12*‐KO, and their respective controls, root tips (up to 5 mm above the root tip) were collected from plants grown in water at 28°C for 2 wk for RT‐qPCR analysis.

To analyze the expression of *ZmSPL12* and *ZmYUC5* genes under osmotic stress, root tips were collected from the WT plants grown in water at 28°C for 1 wk and treated with 15% (w/v) PEG6000 for 5 d for RT‐qPCR analysis; the root tips of plants treated with sterile water were collected at the same time as a control.

To analyze the response of *ZmSPL12* and *ZmYUC5* genes to IAA treatment, root tips were collected from plants grown in water at 28°C for 1 wk treated with 1, 2, 5, 7.5, 10, 25, 50, or 100 nM IAA for 12 h for RT‐qPCR analysis, using root tips from plants treated with sterile water as the control.

### Dual luciferase detection in maize protoplasts

Protoplasts were extracted from the leaves of corn seedlings cultured in a dark room for 2 wk. The free P2YN vector and the vector pGreen linked to the *ZmYUC5* promoter were jointly converted into the control group, whereas the vector P2YN linked to the ZmSPL12 protein and the vector pGreenII 0800‐Luc linked to the *ZmYUC5* promoter were jointly converted into the experimental group. Finally, the detection was carried out using a dual‐luciferase detection kit 11402ES60 (Yeasen Biotechnology Shanghai Co. Ltd, Shanghai, China).

### DAP‐seq analysis

The full‐length coding sequence of *ZmSPL12* was cloned into the Halo‐Tag vector to produce the recombinant GST‐ZmSPL12 for DAP‐seq analysis and then delivered to a commercial sequencing service (Guangzhou Kidio Biotechnology Co. Ltd, Guangzhou, Guangdong, China). The company has classified the sequencing results in detail, revealing downstream genes regulated by ZmSPL12, conserved binding motifs of ZmSPL12 to the promoters of these genes, and potential direct ZmSPL12 target genes (Supporting Information Table S1). A Gene Ontology (GO) (online website: https://systemsbiology.cau.edu.cn/agriGOv2/) enrichment analysis was conducted using the list of target genes whose promoters were bound by ZmSPL12. Combine its returned results with other molecular biology methods to identify downstream promoters that ZmSPL12 may regulate.

### RNA *in situ* hybridization

B73 seedlings were grown under water conditions for 20 d; their root tips were collected and fixed (FAA fixative: anhydrous ethanol: glacial acetic acid: 37% formaldehyde solution: deionized water=5 : 0.5 : 1 : 3.5) for RNA *in situ* hybridization analysis. The antisense probe for *ZmSPL12* was synthesized in the laboratory (Amplification probe 150 bp was cloned onto pEASY‐T vector, and *in vitro* transcription was performed using T7 and SP6 polymerases and UTP labeled with digoxin to obtain sense and antisense RNA probes containing digoxin labeling); the probe and the fixed root tip samples were submitted to Wuhan Servicebio Technology Co. Ltd for hybridization.

### Yeast one‐hybrid (Y1H) assay

The full‐length coding sequence of *ZmSPL12* was cloned into the vector pJG4‐5, whereas the promoter fragments from different target genes were amplified from B73 genomic DNA and cloned into the reporter vector placzi‐2μ. The pJG4‐5‐*ZmSPL12* construct and each promoter reporter construct were introduced into the yeast strain EGY48 according to the operating manual of our laboratory (preparation of yeast EGY48 competent cells, transformation, and plating). Positive colonies were selected for growth on synthetic‐defined (SD) medium lacking uracil and tryptophan (SD/−U−T) and respotted on fresh (SD/−U−T) medium containing 20 mg ml^−1^ X‐gal (sigma, 7240‐90‐6).

### Electrophoretic mobility shift assay

Electrophoretic mobility shift assay (EMSA) was performed using an EMSA Kit (EMSA Probe Biotin Labeling Kit; Beyotime Biotechnology, Shanghai, China) according to the user manual. Single‐stranded DNA probes (*c*. 59 bp) containing the GTAC motif in the 5′ promoter of the potential target gene *ZmYUC5* were synthesized with biotin labeling at their 5′ ends and dissolved in TE to a final concentration of 5 μM.

Expression of GST or GST‐ZmSPL12 recombinant protein: First, the cDNA of ZmSPL12 was amplified from maize RNA and constructed into vector PET‐28a. Then, it was transformed into the *Escherichia coli* expression strain BL21 (DE3) and induced to express the protein at low temperature (16°C) for 24 h with 0.1 mM IPTG. The bacterial cells were collected, and the protein was extracted. Next, use the chemiluminescence EMSA kit to detect biotinylated probes (EMSA Probe Biotin Labeling Kit; Beyotime Biotechnology).

All primer and probe sequences employed throughout this manuscript are provided in Table S2.

### GUS assay

The maize inbred line harboring the *DR5:GUS* reporter is maintained in our laboratory (Zhang *et al*., 2022). *DR5:GUS* was crossed with *ZmSPL12*‐OE and *ZmSPL12*‐KO lines. The F_1_ hybrid seeds were germinated in sterile water for 5 d, and then the root system was incubated in GUS staining solution (10 mM Na_2_EDTA, 50 mM NaH_2_PO_4_, 0.1% Triton‐100, 0.5 mM K_3_[Fe(CN)_6_], 0.5 mM K_4_[Fe(CN)_6_], 2 mM X‐Gluc) and photographed under a stereomicroscope (Leica EZ4).

### IAA treatment experiment

IAA was first dissolved in a small amount of 1 M NaOH, then diluted with deionized water and prepared into different concentrations. Seeds of the *ZmSPL12*‐OE2 lines and their WT were sown in pure water on kraft paper for 5 d and then transferred to pure water containing different concentrations of auxin for further growth for 3 d. The primary roots were then examined and measured. Collect seedling root tips treated with pure water or 100 nM IAA, fix them with FAA fixative, and proceed to the next slicing experiment.

### Statistical analysis

All experiments were performed with at least three independent biological replicates. For phenotypic analyses (e.g. root length, survival rate, yield‐related traits), each replicate consisted of 6–15 individual plants as indicated in the figure legends. For RT‐qPCR and hormone quantification, three technical replicates were performed per biological sample, and their means were used for statistical analysis. In all figures, data are presented as means ± SD. Comparisons between two groups were performed using a two‐tailed Student's *t*‐test. No data points were excluded. Effect sizes (e.g. Cohen's *d* for *t*‐test) are reported in Table S3. Statistical significance was defined as *P* < 0.05.

### Root growth increment ratio

The calculation method for the root growth increment ratio is to divide the difference between the length of treated roots and the length of roots under normal conditions by the length of roots grown under normal cultivation.

## Results

### ZmSPL12 positively regulates root elongation under DS

We previously reported that overexpression of *ZmSPL12* confers enhanced root penetration in compact soils (Xu *et al*., 2024). As drought can harden soils (Lynch *et al*., 2014), we wondered whether *ZmSPL12* might also contribute to drought resistance in maize. To test this idea, we first examined the response of *ZmSPL12* expression to PEG (Fig. S1a). Quantitative analysis revealed that *ZmSPL12* expression is significantly induced in root tissues when exposed to osmotic stress, indicating its likely involvement in mediating DS adaptation.

Next, we conducted stress experiments, using PEG treatment to simulate drought. The root systems of *ZmSPL12*‐OE seedlings cultivated in a hydroponic system without PEG were shorter than those of the control lines; in contrary, the root systems of *ZmSPL12*‐OE seedlings were significantly longer than those of the control under PEG treatment (Fig. S1b–d). Consistently, *ZmSPL12*‐OE seedlings were more resistant to simulated DS than the corresponding nontransgenic control seedlings, based on the extent of leaf wilting and yellowing (Fig. S1e).

To validate the differential root elongation phenotype observed under osmotic stress, we conducted soil‐based drought experiments and quantified the survival rate of the *ZmSPL12*‐overexpression lines (*ZmSPL12*‐OE1, *ZmSPL12*‐OE2), *ZmSPL12* KO lines (*Zmspl12‐*1, *Zmspl12‐*2), and their respective WT controls (Fig. 1a,c). Consistent with the results in the hydroponic setting, the soil‐grown *ZmSPL12‐*OE seedlings had shorter primary roots than their controls under WW conditions but exhibited significantly greater root growth increment ratio under DS conditions. Consistently, the *ZmSPL12‐*KO lines showed the opposite pattern, with longer roots than their controls under WW conditions but less root growth increment ratio under DS conditions (Fig. 1b,d,e). The observed drought‐induced relative greater root elongation in the *ZmSPL12‐*OE seedlings likely represents an acclimation strategy to enhance water acquisition from deeper soil layers in response to DS, suggesting that *ZmSPL12* is a positive regulator of primary root elongation specifically under drought conditions.

**Fig. 1:**
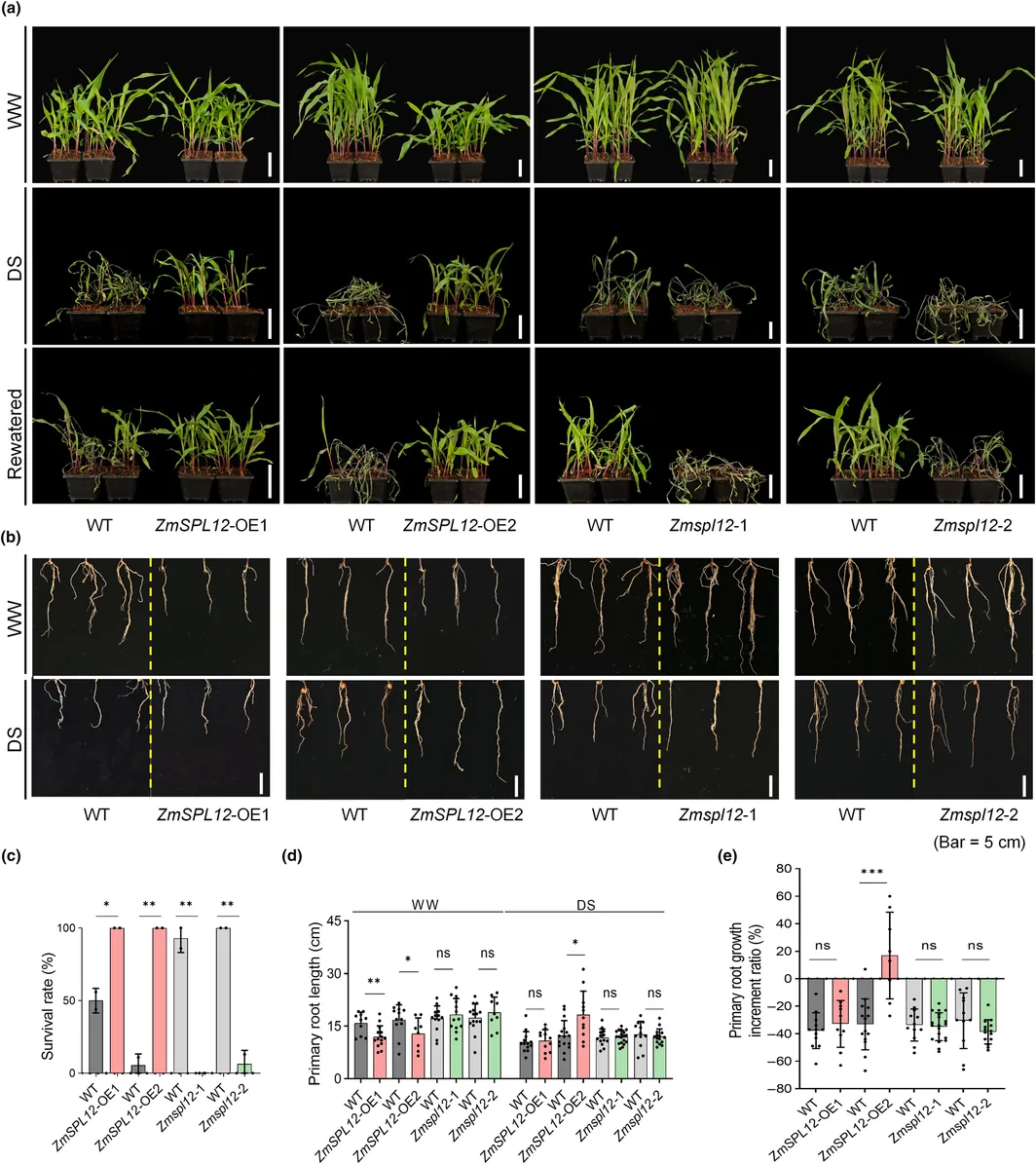
*ZmSPL12* positively regulates primary root elongation of maize seedlings under drought stress (DS). (a) Representative photographs of seedlings from the *ZmSPL12‐*OE (*ZmSPL12‐*OE1, *ZmSPL12‐*OE2) lines and their wild‐types (WTs), the *ZmSPL12‐*KO (*Zmspl12*‐1, *Zmspl12‐*2) lines and their WTs under well‐watered (WW) conditions (top panel), DS conditions (middle panel) for 20 d, and after rewatering for 3 d (bottom panel). Bars, 5 cm. (b) Representative photographs of 23‐d‐old seedlings from the *ZmSPL12‐*OE lines, the *ZmSPL12‐*KO lines, and their WTs, showing root length under WW (upper panel) and DS (lower panel) conditions. Bars, 5 cm. (c) Survival rate following 3 d of rewatering after DS treatment (*n* = 3 biological replicates). (d) Primary root length of *ZmSPL12*‐OE lines, *ZmSPL12*‐KO lines, and their WTs under WW and DS conditions (*n* = 6 plants). (e) Primary root growth increment ratio of *ZmSPL12*‐OE lines, *ZmSPL12‐*KO lines, and their WTs under DS conditions (*n* = 3 biological replicates). In (c–e), data represent mean ± SD. Statistical significance of differences was determined by a two‐tailed Student's *t*‐test. The asterisks indicate significant differences compared to the WT: ns, not significant; *, *P* < 0.05; **, *P* < 0.01; ***, *P* < 0.001.

### ZmSPL12 enhances drought resistance and improves yield stability under water‐limited conditions

Drought represents a major constraint to maize productivity and yield (Li *et al*., 2024). To assess whether *ZmSPL12*‐OE alleviates drought in the field by regulating root growth increment ratio and to evaluate its agronomic potential, we conducted field trials using *ZmSPL12*‐OE, *ZmSPL12*‐KO, and their respective controls under WW and severe DS conditions. Plants were grown in a rain‐off shelter equipped with a retractable roof and controllable irrigation to simulate realistic field environments and precisely control volumetric water content (VWC) (Fig. S2a). Under WW conditions, soil VWC was maintained at *c*. 30% throughout the maize growth cycle. Drought treatment was initiated at 40 d after sowing and sustained until the R1 stage (Bergstrom *et al*., 1987; Sharp *et al*., 2004), with VWC maintained at *c*. 10% under DS conditions (Fig. S2b). Representative images demonstrate that under severe DS conditions, the *ZmSPL12*‐OE lines exhibited significantly higher yield compared to the control lines, whereas the *ZmSPL12*‐KO lines showed reduced yield performance (Figs 2a, S2c).

**Fig. 2:**
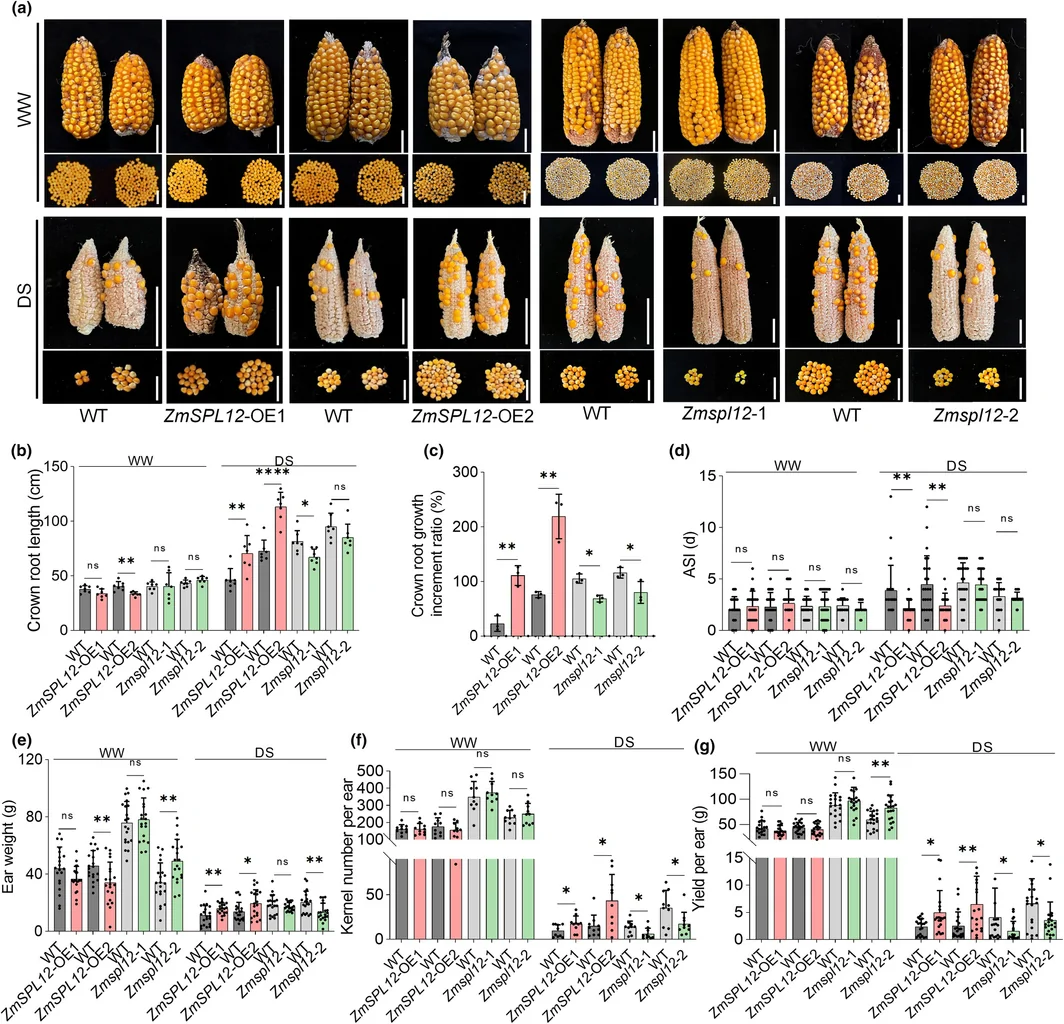
Overexpression of *ZmSPL12* in maize enhances drought resistance. (a) Representative photographs of the ears obtained from the *ZmSPL12*‐OE lines (left), the *ZmSPL12*‐knockout (KO) lines (right) and their wild‐types (WTs) when grown under well‐watered (WW) (upper panel) or severe drought stress (DS) (lower panel) conditions. Bars, 3 cm. (b) Crown root length of the *ZmSPL12‐*OE lines, *ZmSPL12*‐KO lines, and their WTs under WW and severe DS conditions in the field. (c) Crown root growth increment ratio of the *ZmSPL12‐*OE lines, *ZmSPL12*‐KO lines, and their WTs under severe DS conditions, normalized to that under WW conditions. (d–g) Anthesis‐silking interval (ASI) (d) (*n* = 25 plants), ear weight (e) (*n* = 20 plants), kernel number per ear (f) (*n* = 20 plants), and yield per ear (g) ear (*n* = 20 plants) of the *ZmSPL12‐*OE lines, *ZmSPL12*‐KO lines, and their WTs under WW and severe DS conditions in the field. In (b–g), values are means ± SD, with individual data points shown as black circles. Statistical significance of differences was determined by a two‐tailed Student's *t*‐test. The asterisks indicate significant differences compared to the WT: ns, not significant; *, *P* < 0.05; **, *P* < 0.01; ****, *P* < 0.0001.

To elucidate the mechanism underlying drought resistance in the *ZmSPL12*‐OE plants, we first measured leaf water content in mature leaves. Results showed no significant differences in leaf moisture among Z*mSPL12*‐OE, Z*mSPL12*‐KO, and their respective WTs under either WW or DS conditions (Fig. S2d), suggesting stomatal regulation may not account for the enhanced drought resistance of the *ZmSPL12*‐OE plants. We next investigated root system phenotypes. Given that crown roots serve as the primary water‐absorbing organs in mature maize plants (Ahmed *et al*., 2018), we focused our analysis on this root type. Crown roots are typically considered to have ceased elongation and growth by the R1 stage (Bergstrom *et al*., 1987; Sharp *et al*., 2004). We therefore investigated the root phenotype of *ZmSPL12*‐OE, Z*mSPL12*‐KO, and their WTs grown under field conditions at the R1 stage (Fig. S3a,b). Under WW conditions, the crown root length of *ZmSPL12*‐OE lines and their controls were comparable. However, under severe DS conditions, the OE lines displayed significantly greater root elongation ratio (Fig. 2b,c). No significant differences in other root architectural traits were observed, including root diameter, brace root number, crown root number, and brace root layer number, between the *ZmSPL12*‐OE, *ZmSPL12*‐KO, and control lines (Fig. S4a–d). These findings suggest that *ZmSPL12* specifically promotes root elongation under DS conditions without altering overall root morphology.

As a monoecious plant, maize relies on the synchronous development of male and female inflorescences for optimal grain yield. DS during flowering often disrupts this synchrony, leading to an extended ASI and substantial yield losses (Liu *et al*., 2021). To assess the role of *ZmSPL12* in reproductive resilience, we measured ASI in the transgenic and control lines. Under DS conditions, *ZmSPL12*‐OE plants exhibited significantly shorter ASIs than controls (Fig. 2d), indicating improved reproductive synchrony under stress. This phenotypic advantage translated into tangible yield benefits: the OE lines produced heavier ears (Fig. 2e) with more kernels (Fig. 2f), ultimately achieving a 75.0% higher yield per ear under severe DS compared to controls (Fig. 2g). Consistent with these observations, silk number and length showed no significant variation among *ZmSPL12*‐OE, *ZmSPL12*‐KO, and control plants under WW conditions. However, under severe DS, the *ZmSPL12*‐OE plants developed longer and more abundant silks, whereas the *ZmSPL12*‐KO plants produced fewer silks than controls (Fig S5a,b). Thus, we speculate that drought‐enhanced root elongation in the *ZmSPL12*‐OE plants likely improves water uptake capacity, thereby sustaining silk growth and ultimately increasing the seed‐setting rate under water‐limited conditions.

### ZmSPL12 modulates auxin signaling to promote drought‐acclimating root elongation

To systematically characterize the root elongation phenotype of *ZmSPL12*‐OE under DS conditions, we subjected 5‐d‐old seedlings of *ZmSPL12*‐OE2, *Zmspl12‐1*, and their corresponding WT controls to two treatment regimes, continuous osmotic stress (15% PEG6000 aqueous solution) and standard culture conditions (water only) for seven consecutive days (Figs 3a, S6a). Under control conditions, the *ZmSPL12*‐OE2 seedlings exhibited significantly shorter roots than the WT, whereas the *Zmspl12*‐1 mutants had longer roots (Fig. S6a,b). Notably, the genotype‐dependent differences in root length observed under control conditions gradually diminished under PEG treatment (Fig. 3a,b). Consistent with soil‐based phenotypes, the *ZmSPL12*‐OE2 seedlings showed a 6.0% higher root growth increment ratio, whereas the *Zmspl12*‐1 mutants exhibited a 15.6% reduction in root growth increment ratio relative to the WT under osmotic stress (Fig. S6c,d).

**Fig. 3:**
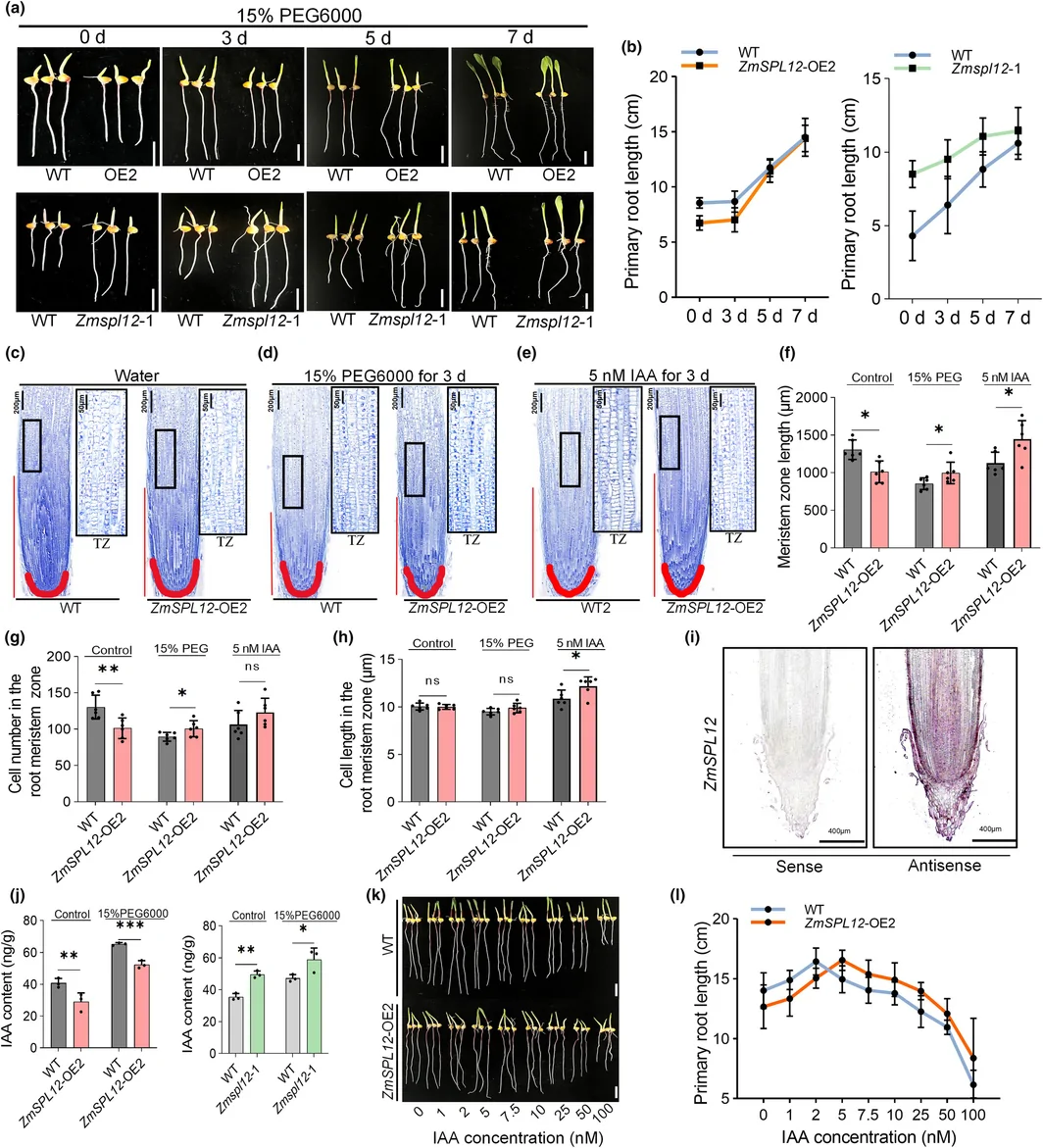
Overexpression of *ZmSPL12* in maize mitigates auxin‐mediated growth inhibition under drought stress (DS). (a) Representative photos of 5‐d‐old *ZmSPL12*‐OE2 and its wild‐type (WT) (upper panel), *Zmspl12‐*2 and its WT (lower panel) treated with 15% PEG6000 for 7 d. Bars, 3 cm. (b) Primary root length of 5‐d‐old *ZmSPL12*‐OE2 and its WT (left) and *Zmspl12‐*2 and its WT (right) treated with 15% PEG6000 for 7 d (*n* = 6 plants). (c) Toluidine blue staining on histological sections of root tips from seedlings grown under normal conditions, showing smaller meristems in *ZmSPL12*‐OE2 relative to its control. (d) Toluidine blue staining on histological sections of root tips after a 3‐d treatment with 15% PEG6000, demonstrating attenuated growth inhibition in *ZmSPL12*‐OE2 relative to its control. (e) Toluidine blue staining on histological sections of root tips after a 3‐d treatment with 5 nM IAA, demonstrating enhancement growth in *ZmSPL12‐*OE2 relative to its control. (f) Lengths of the meristematic regions in the root tips of *ZmSPL12*‐OE2 and its WT seedlings under normal conditions, 15% PEG6000 treatment, 3‐d 5 nM IAA treatment (*n* = 3 biological replicates). (g) Cell numbers in the meristematic regions of root tips from *ZmSPL12‐*OE2 and its WT under normal conditions, 15% PEG6000 treatment, 3‐d 5 nM IAA treatment (*n* = 3 biological replicates). (h) Cell length in the meristematic regions of root tips from *ZmSPL12‐*OE2 and its WT under normal conditions, 15% PEG6000 treatment, 3‐d 5 nM IAA treatment (*n* = 3 biological replicates). (i) RNA *in situ* hybridization localizing *ZmSPL12* transcripts in root tips. The sense probes served as controls. Bars, 400 μm. (j) IAA levels in the root tips of *ZmSPL12*‐OE2 and its WT (left), *Zmspl12‐2*, and its WT (right) under WW conditions or after 5 d of 15% PEG6000 treatment (*n* = 3 biological replicates). (k) Representative photos of *ZmSPL12‐*OE2 and its WT under different IAA concentrations (0, 1, 2, 5, 7.5, 10, 25, 50, or 100 nM). Bars, 3 cm. (l) Primary root length of *ZmSPL12‐*OE2 and its WT under different IAA concentrations treatment (0, 1, 2, 5, 7.5, 10, 25, 50, or 100 nM). In (f–i), values are means ± SD with individual data points shown as circles. Statistical significance of differences was determined by a two‐tailed Student's *t*‐test. The asterisks indicate significant differences compared to the WT: ns, not significant; *, *P* < 0.05; **, *P* < 0.01; ***, *P* < 0.001.

To understand the cellular basis of the root shortening in *ZmSPL12*‐OE, we performed histological sections of root tips. The meristematic zone in *ZmSPL12*‐OE2 was 22.3% shorter (1013.8 ± 132.3 μm vs 1306.2 ± 119.0 μm; *P* < 0.05) and contained 22.3% fewer cortical cells (101.3 ± 12.9 vs 130.5 ± 12.1; *P* < 0.01) compared to the WT, but there was no significant difference in cell length (Fig. 3c,f,g,h). For the elongation zone, both its total length and the length of individual elongated cells showed a decreasing trend in *ZmSPL12*‐OE2 relative to the WT; these changes did not reach statistical significance (Fig. S6e–g).

The phytohormone auxin is known to play a critical role in maintaining meristematic activity and cell division (Dello Ioio *et al*., 2008; Di Mambro *et al*., 2017; Yuan *et al*., 2020; Tu *et al*., 2024; Wang *et al*., 2025). RNA *in situ* hybridization localized *ZmSPL12* transcripts specifically to the root tip (Fig. 3i)—a key site of auxin synthesis (Ljung *et al*., 2005; Brumos *et al*., 2018). Thus, we next investigated whether the observed root phenotypes of *ZmSPL12*‐OE2 were linked to alterations in auxin levels. We quantified IAA content in root tips. Under normal conditions, *ZmSPL12*‐OE2 root tips exhibited 34.0% lower IAA content than the WT (*P* < 0.01), whereas *Zmspl12‐1* mutants showed a 35.4% increase (*P* < 0.01) (Fig. 3j). Exogenous IAA application restored root length in the *ZmSPL12*‐OE2 to WT levels, indicating that the shortened root phenotype results from reduced auxin levels (Fig. S6h,i). Under osmotic stress (3 d of 15% PEG6000), IAA levels were significantly upregulated in all genotypes (1.5–2.3‐fold, *P*<0.01), though relative differences between genotypes were maintained (Fig. 3j). The WT roots accumulated more IAA than the *ZmSPL12*‐OE2 under stress (Fig. 3j). Intriguingly, under both high‐concentration IAA and PEG treatment, *ZmSPL12*‐OE2 displayed a higher root growth increment ratio than the WT, with similar anatomical changes (Fig. 3d,e), supporting the idea that altered auxin homeostasis underlies the drought response (Sharma *et al*., 2015; Musazade *et al*., 2025).

To further dissect this dose–responsive relationship, we treated seedlings with varying IAA concentrations. While > 2 nM IAA inhibited root elongation in the WT, it continued to promote elongation in *ZmSPL12*‐OE2 (Fig. 3k,l), indicating that *ZmSPL12*‐OE roots were less sensitive to auxin‐mediated growth inhibition. At 5 nM IAA, the WT seedlings showed a 31.2% greater reduction in meristem length, a 14.3% greater loss in cortical cell number, and a 12.7% reduction in cell length compared to *ZmSPL12*‐OE2 (Fig. 3e–h). Importantly, under PEG stress, *ZmSPL12*‐OE2 roots showed enhanced cell division and expansion in the meristematic zone compared to the WT (Fig. 3d,f,g), reinforcing that auxin plays a critical role in mediating these adaptive responses.

Together, these results indicate that *ZmSPL12* negatively regulates basal auxin levels in the root tips, leading to shorter roots under normal conditions, while DS elevates IAA content across genotypes, but the lower baseline in *ZmSPL12*‐OE allows greater growth plasticity and higher root growth increment ratio under stress. These findings position *ZmSPL12* upstream of auxin signaling pathways that coordinate root growth under water deficit.

### ZmSPL12 binds to the *ZmYUC5* promoter and represses its expression

To investigate the molecular mechanisms by which ZmSPL12 mediates drought‐induced root growth, we performed DNA affinity purification sequencing (DAP‐seq) to identify its potential target genes (O'Malley *et al*., 2016). The two biological replicates revealed 14 658 common peaks in genic regions (Fig. S7a). We assigned each peak to a genomic feature, which showed that 45.2% of the peaks are located in distal intergenic regions, another 16.4% in promoters, 8.6% in downstream regions, and 6.0% in 3′ untranslated regions (3′ UTRs) (Fig. 4a). MEME‐ChIP motif analysis identified a highly significant GTAC motif (*E*‐value = 8.7e−31) within the ZmSLP12‐bound peaks (Fig. S7b), consistent with known binding sites for SPL members (Wang *et al*., 2009; Wang & Wang, 2015).

**Fig. 4:**
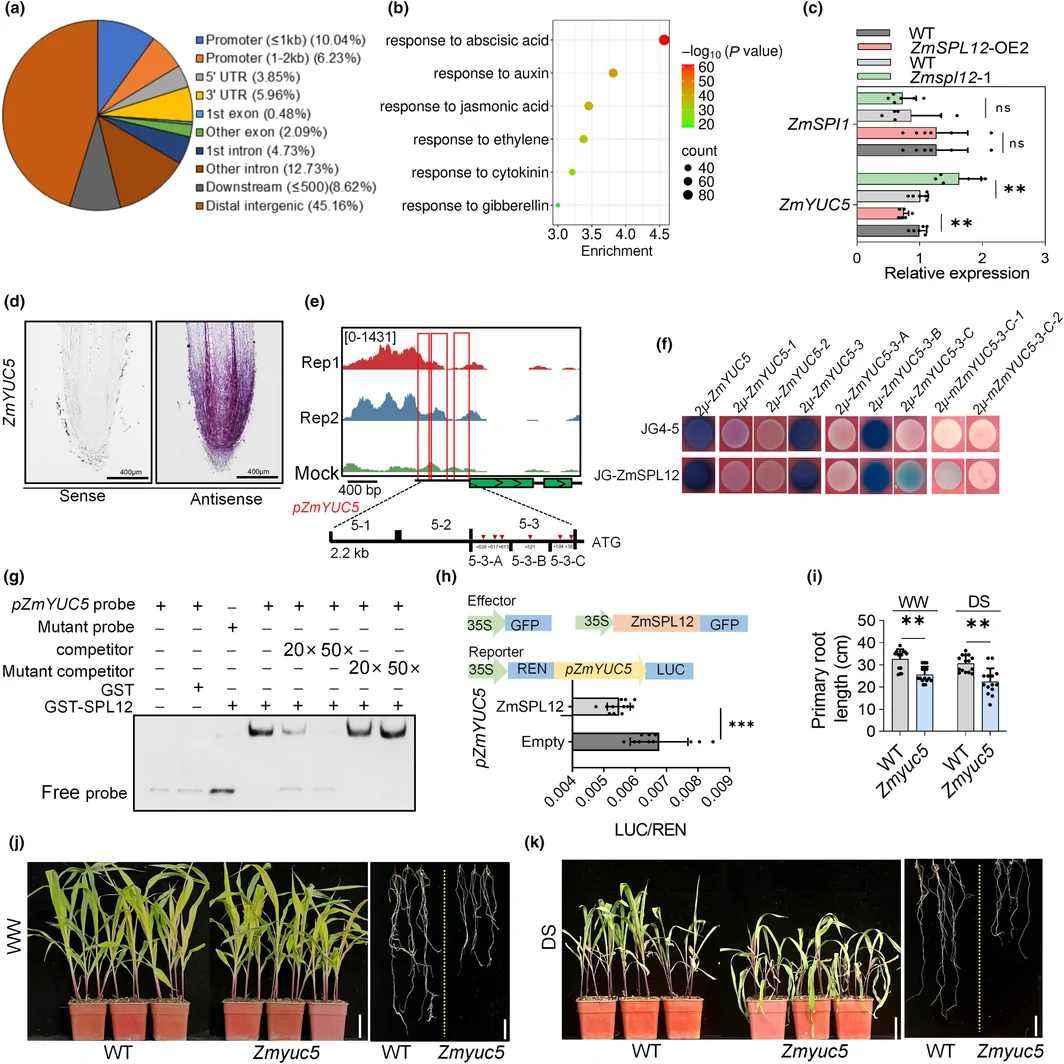
*ZmYUC5* is a potential target of ZmSPL12 in maize. (a) Distribution of ZmSPL12‐binding sites among genomic features, as identified by DAP‐seq. (b) Gene Ontology term enrichment analysis for phytohormone‐related pathways among ZmSPL12 target genes. The *x*‐axis shows the gene ratio (ZmSPL12 target genes in each pathway/total annotated genes). Circle size represents gene count; color indicates −log_10_(*P*‐value). (c) Reverse transcription‐quantitative polymerase chain reaction analysis of *ZmYUC5* and *ZmSPI1* expression levels in root tips of *ZmSPL12*‐OE2, *Zmspl12‐*2, and their wild‐types (WTs)s after 2 wk of cultivation (*n* = 3 biological replicates). (d) RNA *in situ* hybridization localizing *ZmYUC5* transcripts in root tips. The sense probes served as controls. Bars, 400 μm. (e) Genome browser view of ZmSPL12 DAP‐seq binding signals on the ZmYUC5 promoter. The upper panel exhibits DAP‐seq signals from two independent biological replicates (Rep1, Rep2), with discrete red boxes marking three primary truncated promoter fragments designated 5–1, 5–2, and 5–3. Red dots distributed in domains A, B, and C below indicate GTAC *cis*‐elements bound by ZmSPL12. These three primary truncation regions correspond to the constructs tested in (f), which includes additional subtruncations 5‐3‐A, 5‐3‐B, 5‐3‐C, 5‐3‐C‐1, and 5‐3‐C‐2. The subfragments each contain the genomic regions marked by GTAC red dots in this figure. A 2.2 kb sequence upstream of the ZmYUC5 ATG start site is labeled at the bottom. (f) Yeast one‐hybrid (Y1H) assay demonstrating that ZmSPL12 binds to the GTAC motifs in the *ZmYUC5* promoter. (g) Electrophoretic mobility shift assay (EMSA) showing the direct binding of recombinant purified GST‐ZmSPL12 to biotin‐labeled *ZmYUC5* promoter probes. Binding specificity was validated by competition with cold probe. (h) Dual‐luciferase assays confirming the ZmSPL12‐dependent repression of *ZmYUC5* transcription. (i) Primary root length of WT and *Zmyuc5* seedlings under well‐watered (WW) or drought stress (DS) conditions (*n* = 10 plants). (j) Representative photographs (left) and primary root length (right) of 20‐d‐old WT and *Zmyuc5* seedlings under WW conditions. Bars, 5 cm. (k) Representative photographs (left) and primary root length (right) of 20‐d‐old WT and *Zmyuc5* seedlings under DS conditions. Bars, 5 cm. In (c, h, i, j), values are means ± SD with individual data points shown as black circles. Statistical significance of differences was determined by a two‐tailed Student's *t*‐test. The asterisks indicate significant differences compared to the WT: ns, not significant; **, *P* < 0.01; ***, *P* < 0.001.

A GO term enrichment analysis of the putative ZmSPL12 target genes revealed enrichment in biological processes such as ‘response to chemical, plant organ development’, ‘response to phytohormones’, and ‘response to biotic stimulus’ (Fig. S7c). Given the observed shorter roots of the *ZmSPL12*‐OE plants under WW conditions, we investigated a potential link between ZmSPL12 and auxin signaling, using the auxin output reporter *DR5:GUS* driving β‐glucuronidase expression from the synthetic auxin‐responsive *DR5* element (Zhang *et al*., 2022). When the root tips of F_1_ seedlings derived from a cross between the inbred line carrying the *DR5:GUS* reporter and *ZmSPL12*‐OE or *ZmSPL12*‐KO lines were stained for GUS activity, we observed lower GUS staining in the root tips of the *ZmSPL12*‐OE seedlings, but stronger GUS staining in those of the *ZmSPL12*‐KO seedlings, relative to their corresponding controls (Fig. S7d,e). These observations suggest that *ZmSPL12* negatively regulates auxin biosynthesis and/or signaling in the root tips. In support of this idea, multiple GO terms related to phytohormone‐related pathways were enriched in putative ZmSPL12 target genes, including ABA, auxin, and jasmonic acid signaling (Fig. 4b).

By integrating DAP‐seq binding profiles (Fig. 4a,e) with *ZmSPL12* expression patterns (Fig. 3i), we identified the auxin biosynthesis gene *ZmYUC5* and the related gene *sparse inflorescence1* (*ZmSPI1*) as putative direct targets of ZmSPL12. RT‐qPCR assay revealed that ZmSPL12 negatively regulates *ZmYUC5* expression, showing significant downregulation in the *ZmSPL12*‐OE roots and upregulation in the *ZmSPL12*‐KO roots, while *ZmSPI1* expression remained unchanged (Fig. 4c). Spatial expression analysis through RNA *in situ* hybridization localized *ZmYUC5* transcripts specifically to the root meristematic zone (Fig. 4d), corresponding to known sites of auxin production. Molecular validation demonstrated that direct binding of ZmSPL12 to the GTAC *cis*‐element in the *ZmYUC5* promoter via Y1H assay and EMSA, with binding abolished by the GTAC → GCAC mutation (Fig. 4e–g). Functional repression of *ZmYUC5* transcription by ZmSPL12 was further confirmed in *N. benthamiana* leaves (19.0% reduction in luciferase activity) and maize protoplasts (51.9% reduction in luciferase activity) (Figs 4h, S7f).

To validate the functional relationship between *ZmSPL12* and *ZmYUC5* under DS, we conducted gene expression profiling analysis. The results demonstrated coordinated but differential upregulation of *ZmSPL12* (36%, *P* < 0.05) and *ZmYUC5* (178%, *P* < 0.01) under drought, with their expression levels showing a strong positive correlation (*R* ^2^ = 0.54) during stress treatment, indicative of a dose‐responsive co‐regulation of *ZmSPL12* and *ZmYUC5* during drought adaptation (Fig. S7g,h). Moreover, expression of both genes was induced by auxin treatment, with *ZmYUC5* showing a stronger transcriptional elevation (127%) than *ZmSPL12* (40%) under 100 nM IAA (Fig. S7i). This difference may relate to their opposing regulatory functions. As a key auxin biosynthetic gene, *ZmYUC5* is prone to robust activation upon auxin accumulation, while the weaker induction of the repressor ZmSPL12 might act to balance auxin production without complete inhibition, though additional experiments are needed to confirm this hypothesis. These results suggest that ZmSPL12 likely promotes drought‐acclimating root elongation by downregulating *ZmYUC5* expression.

### *ZmYUC5* is essential for root development and drought resistance

To further assess the role of *ZmYUC5* in drought resistance, we characterized a *ZmYUC5* KO mutant line (*Zmyuc5*) generated via CRISPR/Cas9‐mediated genome editing in the C01 inbred background. Sequencing confirmed that the mutants carried deletions in the second and third exons, leading to a truncated protein (Figs S3b, S8a). Under WW conditions, the *Zmyuc5* seedlings exhibited significantly shorter primary roots (reduced by 20.6% ± 5.6%, *P* < 0.05) compared to WT controls (Fig. 4i,j), a phenotype consistent with that of *ZmSPL12*‐OE plants. However, unlike the drought‐resistant *ZmSPL12*‐OE lines, the *Zmyuc5* displayed marked drought sensitivity, with significantly reduced survival rates (75.4% ± 5.4% vs 90.8% ± 9.2% in WT, *P* < 0.05) following drought/rehydration treatment (Figs 4k, S8c,d). In the field, *Zmyuc5* plants also showed reduced plant height (Fig. S8e,f).

Closer examination of crown root phenotypes revealed that under WW conditions, the root systems of *Zmyuc5* plants failed to develop fully, with multiple root tips showing structural damage (Fig. S8h,i). Although a subset of roots managed to elongate, likely, compensating for water uptake, the overall root system remained underdeveloped (Fig. S8g,j). Under DS conditions, despite the upregulation of *ZmYUC5* expression in WT plants (Fig. S7h), the *Zmyuc5* roots only partially recovered, resulting in a root system still weaker than that of the WT and poor drought resistance overall (Fig. S8g).

These results indicate that *ZmYUC5* is essential for normal root elongation under standard growth conditions. Unlike the fine‐tuning role of ZmSPL12 in modulating auxin levels, loss of *ZmYUC5* directly impairs root development, and this impairment cannot be fully reversed under drought. This finding is consistent with previous reports that overexpression of *YUC* family genes enhances both biomass and drought resistance (Park *et al*., 2019; Musazade *et al*., 2025), underscoring that robust growth capacity is a prerequisite for drought resilience.

### Maize hybrids overexpressing *ZmSPL12* show improved drought resistance and yield performance

To translate the observed molecular function of *ZmSPL12* into agricultural applications, we developed improved versions of the elite inbred lines Jing724 and Jing92 (the parental lines of Jingke968, a leading commercial hybrid cultivar in Chinese maize production) through backcross breeding with *ZmSPL12*‐OE transgenic lines (Zhao *et al*., 2022). The material is planted in Urumqi, Xinjiang (Fig. S9). It was watered normally in the first month, and from the second month onward, under DS, the watering amount was half of the normal watering until harvest. The watering amount and rainfall are shown in Tables S4 and S5. Field evaluations of these improved elite inbred lines, named Jing724^OE2^ and Jing92^OE2^, demonstrated significant improvements in drought resistance and yield performance relative to inbred lines Jing724 and Jing92. Under WW conditions, the improved inbred lines produced higher yields, with Jing724^OE2^ showing 18.0% and 6.4% higher yield per ear and 100‐kernel weight, respectively, than Jing724 (*P* < 0.01), while Jing92^OE2^ had 21.2% and 9.1% higher yield per ear and hundred‐kernel weight, respectively, than Jing92 (*P* < 0.01). Importantly, under DS conditions, the improved lines displayed 11.4–41.0% greater values for key yield‐related traits including ear weight, kernel number per row, and kernel number per ear (*P* < 0.01), with the overall yield advantage rising to 25.7–41.0% relative to their parental lines (Fig. S10).

To evaluate the potential contribution of *ZmSPL12* to hybrid performance, we used two cross combinations – improved female × male parent (Jing724^OE2^ × Jing92) and female × improved male parent (Jing724 × Jing92^OE2^)—with F_1_ hybrids derived from a cross between the two parental lines (Jing724 × Jing92) serving as controls (Fig. 5a). Under WW conditions, there was no significant difference in the number of rows number per ear between the improved male or female parent, but there were significant differences in the number of kernels per row and ear weight. Under DS conditions, we observed distinct parent‐of‐origin effects: F_1_ hybrids derived from an improved male parent produced significantly more rows per ear (+7.1%), more kernels per row (+11.6%), and heavier ears (+37.7%). This advantage demonstrates that improving either parent can enhance hybrid drought resistance, but introgression of *ZmSPL12*‐OE through the male parent confers a more pronounced yield benefit under water‐limited conditions (Fig. 5b–d). A clear parent‐of‐origin effect was observed, wherein the improved male parent contributed more significantly to enhancing drought resilience in the hybrid offspring. The mechanistic basis for this phenomenon will be explored in future studies.

**Fig. 5:**
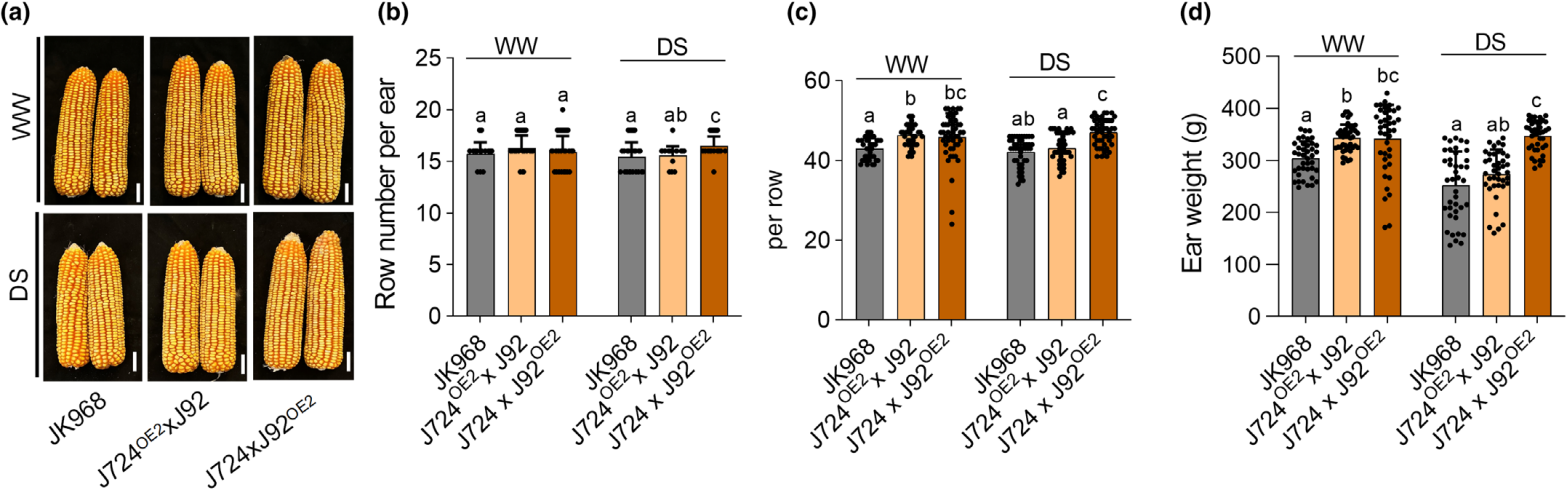
*ZmSPL12*‐overexpressing hybrid lines exhibit enhanced drought resistance in maize under field conditions. (a) Representative photographs of full ears from improved hybrid lines and their controls following growth under well‐watered (upper panel) or moderate DS (lower panel) conditions. Bars, 3 cm. (b–d) Evaluation of yield‐related traits: row number per ear (b), number of kernels per row (c), and ear weight (d) (*n* ≥ 40 plants). In (b–d) values are means ± SD, with individual data points shown as black circles. Statistical significance of differences was determined by a two‐tailed Student's *t*‐test. Different lowercase letters above the bars indicate statistically significant differences between groups (*P* < 0.05). Bars with the same letter are not significantly different.

## Discussion

In this study, we demonstrate that the transcription factor ZmSPL12 acts as a central regulator of drought‐induced root elongation in maize by directly repressing the auxin biosynthesis gene *ZmYUC5*. Under WW conditions, ZmSPL12 binds to the GTAC motif in the *ZmYUC5* promoter, suppressing its expression and maintaining a lower auxin baseline in the root tip, leading to constitutively shorter roots with reduced meristematic size and cortical cell number. DS triggers a universal increase in IAA content across genotypes, the *ZmSPL12*‐OE lines, starting from a lower baseline, exhibit a greater root growth increment ratio, thus allowing them to maintain meristematic activity and cell elongation when WT root growth is inhibited, ultimately facilitating deeper water foraging and improved drought resistance (Fig. 6). Our model thus posits *ZmSPL12* as a ‘developmental brake’ that fine‐tunes auxin homeostasis, priming the root system for enhanced plasticity and sustained growth under water deficit.

**Fig. 6:**
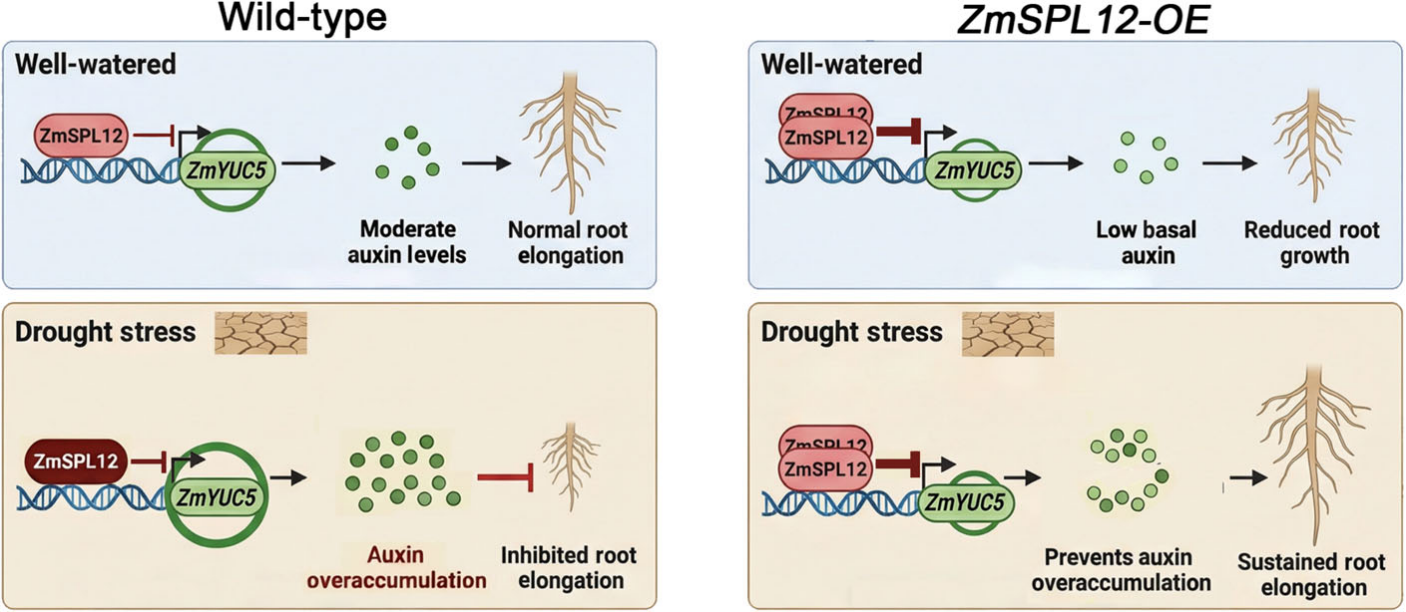
Proposed model of ZmSPL12‐mediated drought resistance through auxin regulation in maize. ZmSPL12 functions as a central regulator of the drought response and is itself induced by DS. In wild‐type plants, drought simultaneously induces both *ZmSPL12* and the auxin biosynthesis gene *ZmYUC5*, which act antagonistically to fine‐tune auxin homeostasis. ZmYUC5 promotes auxin biosynthesis (acting as an ‘accelerator’), whereas ZmSPL12 directly represses *ZmYUC5* (serving as a ‘brake’), together forming a balanced regulatory module. Under well‐watered conditions, this balance maintains moderate auxin levels that support normal root elongation. Under DS, although both genes are induced, the promotive effect of ZmYUC5 predominates, leading to excessive auxin accumulation in root tips, which inhibits root elongation and limits water uptake. In *ZmSPL12*‐overexpressing plants, this regulatory balance is shifted toward repression. Enhanced ZmSPL12 activity strengthens inhibition of *ZmYUC5*, thereby reducing basal auxin levels and preventing drought‐induced auxin overaccumulation. As a result, although root growth is somewhat reduced under normal conditions, these plants maintain sustained root elongation under water deficit, ultimately improving drought adaptation. In the schematic, arrow thickness and inhibitory line weight indicate the strength of activation or repression, respectively. The color intensity of ZmSPL12 represents its expression level, and the circle size surrounding *ZmYUC5* reflects its transcriptional activity.

The YUCCA flavin monooxygenase family constitutes the rate‐limiting core component of the canonical TAA/YUC auxin biosynthetic pathway, which dominates local auxin production in root apical tissues across angiosperms (Brumos *et al*., 2018; Blakeslee *et al*., 2019). Extensive prior genetic evidence in model plants and crops has established the universal function of *YUC* genes in coordinating root development and abiotic stress adaptation. In Arabidopsis, root‐expressed *YUC3/5/7/8/9* are indispensable for apical meristem maintenance. Loss‐of‐function mutations of these loci drastically truncate primary root growth by depleting local IAA pools (Ljung *et al*., 2005). Multiple studies further link YUC‐dependent auxin biosynthesis to drought tolerance, yet conflicting functional models have been reported across species and genotypes. Ectopic overexpression of *AtYUCCA6* and *AtYUCCA7* in Arabidopsis elevates endogenous auxin abundance and confers robust drought resilience via enhanced root biomass and reactive oxygen species scavenging capacity (Lee *et al*., 2012; Cha *et al*., 2015). Consistent observations are replicated in transgenic potato and soybean lines overexpressing homologous *YUC* orthologs (Kim *et al*., 2013; Park *et al*., 2019). In rice, the *YUC* family member *OsCOW1* drives root auxin synthesis, and *cow1* loss‐of‐function mutants display impaired water absorption and constitutive drought hypersensitivity (Woo *et al*., 2007). Recent work in mungbean further confirms that *YUC* genes dynamically adjust cellular IAA concentrations to balance growth and drought acclimation (Wu *et al*., 2023). Notably, nearly all previous work frames YUCs as positive regulators of drought resistance by boosting total auxin levels, while our study reveals a context‐dependent dual regulatory logic specific to maize root tips. WT maize accumulates excessive IAA in root cortical tissues under drought, which restrains meristematic cell division and elongation, a phenotype consistent with earlier anatomical observations in water‐deficient maize roots (Chimungu *et al*., 2014a,b). *ZmYUC5* is the primary contributor to this drought‐triggered auxin overaccumulation in root meristems. Complete KO of *ZmYUC5* (*Zmyuc5*) severely impairs basal root elongation under WW conditions and fails to sustain root growth upon drought exposure (Fig. 4I–K). This distinguishes *ZmYUC5* from stress‐tolerant YUC paralogs characterized in Arabidopsis and legumes. *ZmYUC5* is essential for normal root development, yet its drought‐induced hyperactivation becomes growth‐inhibitory. This trade‐off creates a critical regulatory gap that our ZmSPL12–ZmYUC5 module resolves, ZmSPL12 acts as a transcriptional buffer to constrain excessive *ZmYUC5* induction under water deficit, preventing toxic auxin buildup without eliminating basal auxin biosynthesis required for root viability. This mechanism expands the existing paradigm of YUC‐mediated drought responses by introducing a tissue‐specific negative transcriptional rheostat that calibrates, rather than constitutively elevates, auxin levels for stress adaptation.

Beyond our identified ZmSPL12 transcriptional brake, the plant literature reports multiple classes of ‘developmental brake’ modules that fine‐tune auxin concentration gradients to balance growth and stress tolerance. This fine‐tuning mechanism of auxin homeostasis mediated by transcription factor‐YUC modules has also been documented in legume‐rhizobium symbiosis, a developmental process tightly coupled to auxin gradients. Optimal auxin concentrations, precisely balanced via transcriptional repression of YUC homologs, are required for proper soybean nodule maturation and symbiosome formation (Tu *et al*., 2024; Chen *et al*., 2026). Analogous multi‐hormonal crosstalk networks also converge to modulate auxin biosynthetic flux under environmental stimuli (Semeradova *et al*., 2020). For example, jasmonic acid hormone may help fine tune the flow of auxin by regulating the transport of PIN2 (Sun *et al*., 2011), GA regulates the accumulation of PIN proteins on the plasma membrane, thereby fine tuning the polar transport of auxin (Willige *et al*., 2011), and cytokinin may be important in maintaining the size of root tip meristematic tissue or lateral root organ development through posttranslational fine‐tuning of auxin polar transport mechanism (Marhavý *et al*., 2014; Semeradova *et al*., 2020). In addition, phosphorylation also plays a role in the rapid fine‐tuning of auxin transport in response to external stimuli (Dory *et al*., 2018). Auxin itself forms concentration gradients via coordinated local biosynthesis, degradation, and polar cell‐to‐cell transport, serving as a master signal integrating developmental and environmental cues across all plant organs (Vanneste *et al*., 2025). Multiple hormone signaling cascades converge to modulate auxin metabolism and transport, constructing an intricate signaling network that orchestrates plastic plant growth under fluctuating water availability.

Compared with those previously characterized brake systems, ZmSPL12 represents a tissue‐specific, drought‐activated transcriptional brake that directly targets the rate‐limiting auxin biosynthetic gene *ZmYUC5* in root meristems. Most documented auxin brakes function constitutively throughout development or respond to general developmental aging. Few transcription factors are specifically induced by water deficit to serve as a stress‐tunable rheostat for root‐localized auxin production. This context‐specific ‘conditional brake’ function of ZmSPL12 represents a novel addition to the catalog of auxin homeostatic regulators reported to date.

While auxin has long been implicated in stress responses, a clear molecular pathway connecting drought perception to auxin‐mediated root architectural remodeling has been missing. Our work identifies the ZmSPL12–*ZmYUC5* module as a dedicated drought‐responsive pathway that dynamically regulates root growth. This discovery is novel for several reasons. First, it reveals how a seemingly negative growth regulator under optimal conditions (*ZmSPL12* overexpression shortens roots) can be repurposed to enhance stress resilience, a concept we term ‘conditional plasticity’. Second, we provide a mechanistic link from a drought‐induced transcription factor directly to auxin biosynthesis, bridging a critical knowledge gap. Furthermore, the dose–responsive relationship we uncovered – where *ZmSPL12*‐OE roots are less sensitive to auxin‐mediated growth inhibition – offers a new perspective on how plants can exploit the biphasic nature of auxin signaling to optimize growth under stress. This paradigm shifts the breeding objective from constitutive stress resistance to engineering dynamic, context‐dependent acclimation responses.

Although we have delineated the core ZmSPL12–ZmYUC5–auxin axis, several questions remain. The precise spatiotemporal dynamics of auxin biosynthesis, degradation, and polar transport during drought progression require further elucidation. It has long been suggested that the initial pulse of auxin required for early root development is produced in the buds and transported to the roots through polar auxin transport (Grieneisen *et al*., 2007). As the root develops, a burst of shoot‐derived auxin triggers auxin synthesis in the root apical meristem (Casimiro *et al*., 2001). The highly conserved TAA/YUC pathway is the most clear pathway for auxin biosynthesis. In Arabidopsis, *YUC3*, *YUC5*, *YUC7*, *YUC8*, and *YUC9* are responsible for producing auxin in the roots and mutations in these genes can cause root development to become shorter or not grow (Chen *et al*., 2014). Studies have shown that overexpression of *YUCCA6*, *YUCCA7* gene in Arabidopsis can enhance drought resistance (Lee *et al*., 2012; Cha *et al*., 2015). The research in *Vigna radiata* L. indicates that the YUCCA family participates in growth regulation under abiotic stress by finely regulating IAA content (Wu *et al*., 2023). Some people also believe that the improvement of drought resistance is related to the decrease in IAA content in roots (Du *et al*., 2013; Naser & Shani, 2016). YUCs can also regulate the biosynthesis of local auxin in the root apex TZ, mediating root growth inhibition under abiotic stress (Liu *et al*., 2016; Blakeslee *et al*., 2019). Similarly, the TZ is located between the apical meristem and basal elongation region and is associated with the perception of hormone crosstalk in root growth and development under environmental pressure and stimulation. It is also considered a potential focus for YUC dependent regulation of salt/drought/ion stress responses (Kong *et al*., 2018). The root system maximizes surface area and extracts water from increasingly dry soil (Davies *et al*., 2002), thus some have proposed increasing root biomass/surface area to enhance tolerance, most likely through an increase in lateral root numbers (Moles *et al*., 2018). In this study, we also measured the lateral roots of maize during the seedling and mature stages (Fig. S11). Under WW conditions, the number and length of lateral roots of *ZmSPL12*‐OE decreased, but under DS, the number and length of lateral roots increased significantly. This also suggests that *ZmSPL12*‐OE may increase drought resistance by affecting the number and length of lateral roots, but this mechanism still needs further research. Whether there are other YUC genes mediating drought response, this study obtained the *ZmYUC5* gene by combining DAP‐seq data with the expression pattern of *ZmSPL12*. However, it cannot be confirmed that the DAP‐seq data are completely correct, and it is also possible that other *YUCs* gene are involved, which requires further experiments and phenotype verification. At the same time, we also conducted quantitative experiments on the PIN family of auxin transporters and their response to osmotic stress (Fig. S12). The DAP‐seq data also showed the presence of PIN family proteins, but further experimental confirmation is needed to determine whether ZmSPL12 regulates PIN proteins. Compared to that, we are more interested in the changes in root tips, so we mainly focus on the study of *YUCs* gene. It is unclear how the drought‐induced auxin buildup we observed in root tips coordinates with known transporters like PINs and the auxin minimum in the TZ. Whether *ZmSPL12* is expressed in lateral root primordia, whether ZmSPL12 regulates other downstream targets beyond *ZmYUC5* that contribute to the phenotype, or interacts with other key drought hormones like ABA, remains an open question. The contrasting phenotypes of *ZmSPL12*‐OE and *Zmyuc5* mutants under drought also suggest a more complex network; *ZmYUC5* is essential for normal development, whereas *ZmSPL12* fine‐tunes it. Future work should employ single‐cell transcriptomics and tissue‐specific promoters to dissect this network with higher resolution. Additionally, the striking parent‐of‐origin effect in hybrids, where improving the male parent (Jing92) significantly boosted drought resilience, points to unexplored gametic or epigenetic mechanisms that merit dedicated investigation.

*ZmSPL12* exemplifies a transcriptional integrator that optimally balances developmental programs with environmental cues. Its pleiotropic effects – modulating root growth under drought, root penetration in compacted soil (Xu *et al*., 2024), and lodging resistance (Zhao *et al*., 2022), highlight its role as a master regulator of plant architecture. The breeding value of this gene is substantial. We successfully translated this knowledge by introgressing *ZmSPL12*‐OE into elite inbred lines, which conferred a 25.7–41.0% yield advantage under severe drought without penalizing yield under WW conditions. This addresses a major breeding challenge to improve stress resilience without compromising baseline productivity. The superior performance of F_1_ hybrids derived from the improved male parent underscores the potential of *ZmSPL12* in hybrid breeding programs. Studies have shown that the development and function of pollen are highly sensitive to abiotic stress (Zhang *et al*., 2021), and pollen development involves coordinated meiosis and postmeiosis stages. Even short‐term environmental stress can interfere with pollen vitality and successful fertilization (Begcy & Dresselhaus, 2018; Thajudeen *et al*., 2026). Drought induces male sterility and reduces yield through abnormal another development, therefore maintaining pollen vitality under DS is one of the means to increase yield (J. Yu *et al*., 2019; Lamin‐Samu *et al*., 2021). We speculate that the paternal effect may be related to pollen competitiveness under stress or the stronger influence of the paternal genome during grain filling.

Overall, our study provides both a mechanistic understanding and a validated genetic tool for developing a new generation of ‘smart’ maize varieties that remain high‐yielding under optimal conditions yet dynamically activate resilience mechanisms when confronted with drought.

## Competing interests

None declared.

## Author contributions

MX and HW planned and designed the research. HX and LM performed the experiments. QZ analyzed the data and supplemented the experiments proposed by the reviewer. ZZ and BZ prepared the materials. YZ, JC, YT, JZ, and BW performed phenotypic characterization. LJ, ZB, and JL conducted field pollination. SC and JC determined IAA concentrations. HX and MX wrote the manuscript. HX, QZ, MX, HW, JL, and SY revised the manuscript. HX, LM, and QZ are co‐first authors on this work.

## Disclaimer

The New Phytologist Foundation remains neutral with regard to jurisdictional claims in maps and in any institutional affiliations.

## Supporting information


**Fig. S1** *ZmSPL12* expression is induced in roots under drought stress and promotes root elongation.
**Fig. S2** Plant growth and yield performance under severe drought stress conditions in a rain‐off shelter in the field.
**Fig. S3** Photographs of crown roots under WW and DS conditions.
**Fig. S4** Brace and crown root architectural traits under well‐watered and severe drought stress conditions in the field.
**Fig. S5** Number and length of silks emerging from *ZmSPL12*‐OE lines under drought stress.
**Fig. S6** Overexpression of *ZmSPL12* decreased auxin content.
**Fig. S7** Identification of potential ZmSPL12 direct targets.
**Fig. S8** Plant height and crown root performance in the field of Zmyuc5 and its wild type.
**Fig. S9** Field sowing and emergence situation in Urumqi, Xinjiang.
**Fig. S10** The improved elite parental inbred lines carrying the *ZmSPL12*‐OE transgene show better drought resistance than the parental lines.
**Fig. S11** *ZmSPL12* positively regulates lateral root number and length of maize under drought stress.
**Fig. S12** Differential expression of *ZmPINs* in Zm*SPL12*‐OE2/*Zmspl12‐*2 under normal conditions and in wild type under osmotic stress.

**Table S1** Summary of DAP‐seq peaks for ZmSPL12.
**Table S2** Name and sequence of primers and probes.
**Table S3** Effect sizes for all key comparisons.
**Table S4** Rainfall during the whole growth period of maize in 2024.
**Table S5** Water supply for different moisture treatments (m^3^ per acre).
Please note: Wiley is not responsible for the content or functionality of any Supporting Information supplied by the authors. Any queries (other than missing material) should be directed to the *New Phytologist* Central Office.

## Data Availability

Data supporting the findings of this work are provided in the paper and its Supporting Information file. DAP‐seq peak summaries are provided in Table S1. All primer and probe sequences are listed in Table S2. Effect sizes for statistical comparisons are shown in Table S3. Rainfall and irrigation records for field trials are provided in Tables S4 and S5. Additional phenotypic, expression and histological supporting results are presented in Figures S1–S12. The genetic materials supporting the findings of the current study are available from the corresponding authors upon request. Source data are provided with this paper.
