# Supplementary material for: Coordination of drought‐adaptive root growth plasticity through auxin homeostasis by the ZmSPL12–ZmYUC5 module in maize
Source: New Phytol. 2026 Sep 22;252(4):1526–44. doi: 10.1111/nph.71592 (PMC13669809; doi:10.1111/nph.71592)
Supplement: Supplementary file 1 [file NPH-252-1526-s002.pdf]

New Phytologist Supporting Information

Article title: Coordination of drought-adaptive root growth plasticity through auxin homeostasis by the ZmSPL12–ZmYUC5 module in maize

Authors: Hua Xua,1, Lei Ma a,1, Qingyun Zhanga,c,1, Zhigang Zhengb, Jiajing Lia,c, Sen Yangd, Yanfeng Zhaoa, Lian Jina,c, Binbin Zhaoa,e, Zhijing Baia, Jiyuan Caoa, Youpeng Taoa,c, Junjie Zoua,c, Baobao Wanga, Shujing Chengf,g, Jinfang Chuf,g, Jiafan Liub, Haiyang Wanga,b,*, Miaoyun Xua,b,*

Article acceptance date: 26 August 2026

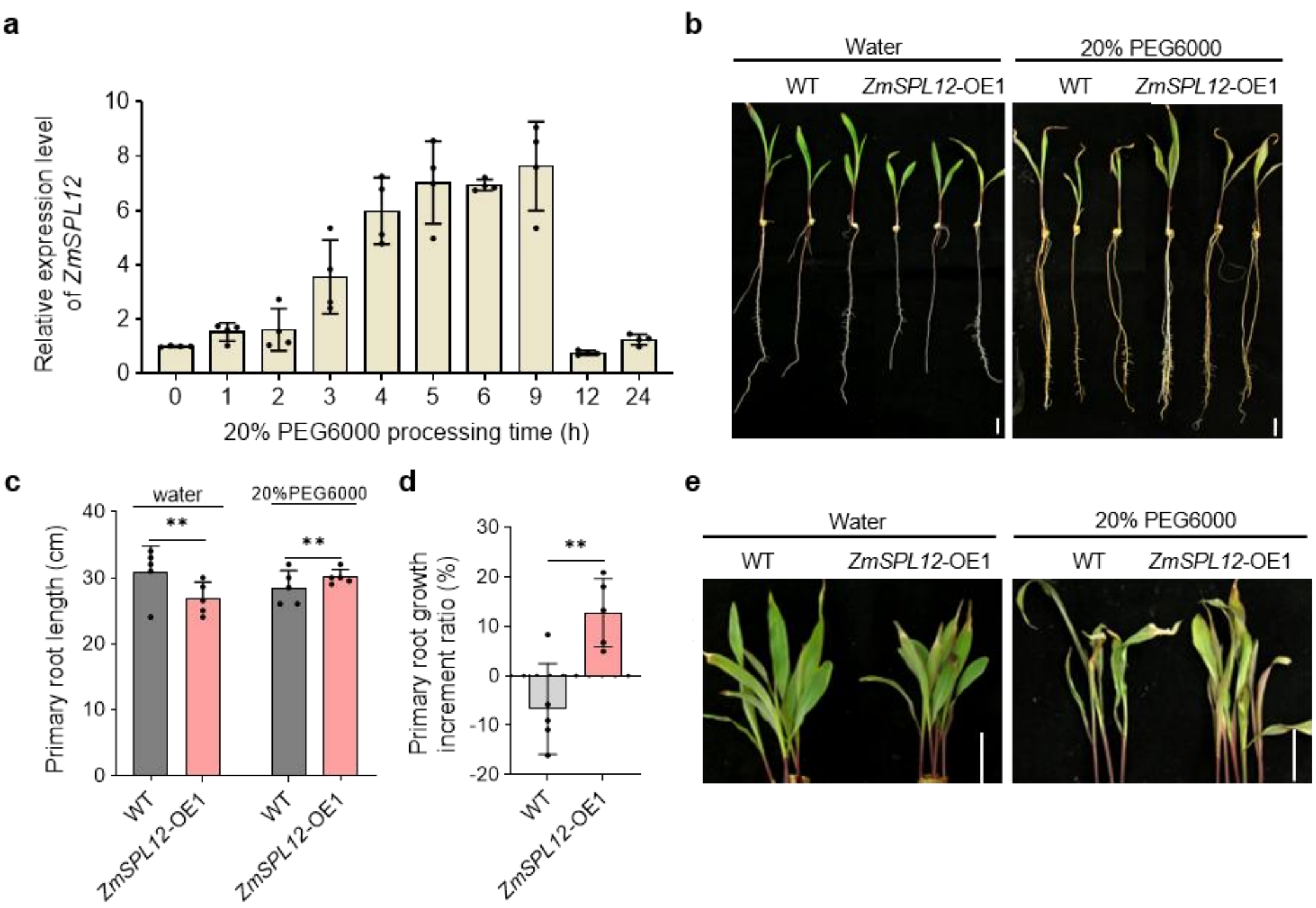

Figure S1. *ZmSPL12* expression is induced in roots under drought stress and promotes root elongation.

a. Relative *ZmSPL12* expression levels under 20% PEG6000 treatment for the indicated times (n = 4 biological replicates). **b**. Representative photos of primary roots of *ZmSP12*-OE1 and its wild type seedlings treated with 20% PEG6000 and hydroponic for 5 days. Scale bars, 3 cm. **c**. Primary root length of *ZmSPL12*-OE1 and its wild type seedlings under WW conditions or 20% PEG6000 treatment (n = 5 plants). **d**. Relative primary root elongation of roots from *ZmSPL12*-OE1 and its wild type seedlings under 20% PEG6000 treatment, normalized to that under WW conditions (n = 5 plants). **e**. Representative photos of *ZmSP12*-OE1 and its wild type seedlings cultured for two weeks followed by 5 days of hydroponic cultivation and 5 days of 20% PEG6000 treatment show the leaf phenotypes under simulated water stress applied under hydroponic conditions or with 20% (w/v) PEG6000 treatment. Scale bars, 3 cm. In **c**–**d**, data represent mean ± s.d. Statistical significance of differences was determined by two-tailed Student's *t*-test. The asterisk indicates significant differences compared to the wild type: **P<0.01.

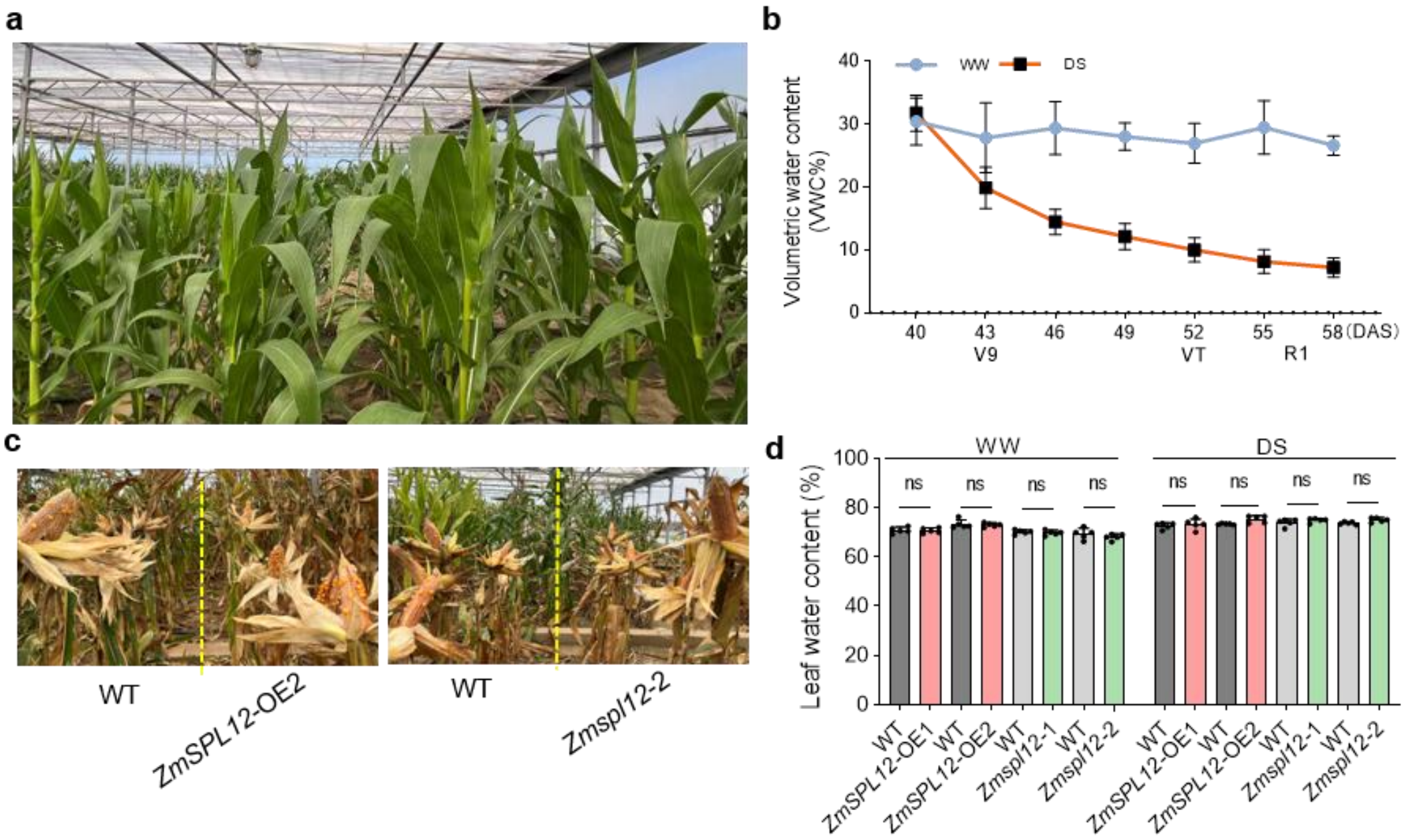

Figure S2. Plant growth and yield performance under severe drought stress (DS) conditions in a rain-off shelter in the field.

a. Photograph of a rain-off shelter in the fields. **b**. VWC under WW and severe DS conditions during plant growth and development. **c**. Photographs of *ZmSPL12*-OE2 (left), *Zmspl12*-2 (right) and their wild types ears harvested from plants growing under severe DS condition. **d**. Leaf water content under WW and DS conditions. In **d**, data represent mean ± s.d. Statistical significance of differences was determined by two-tailed Student's *t*-test.

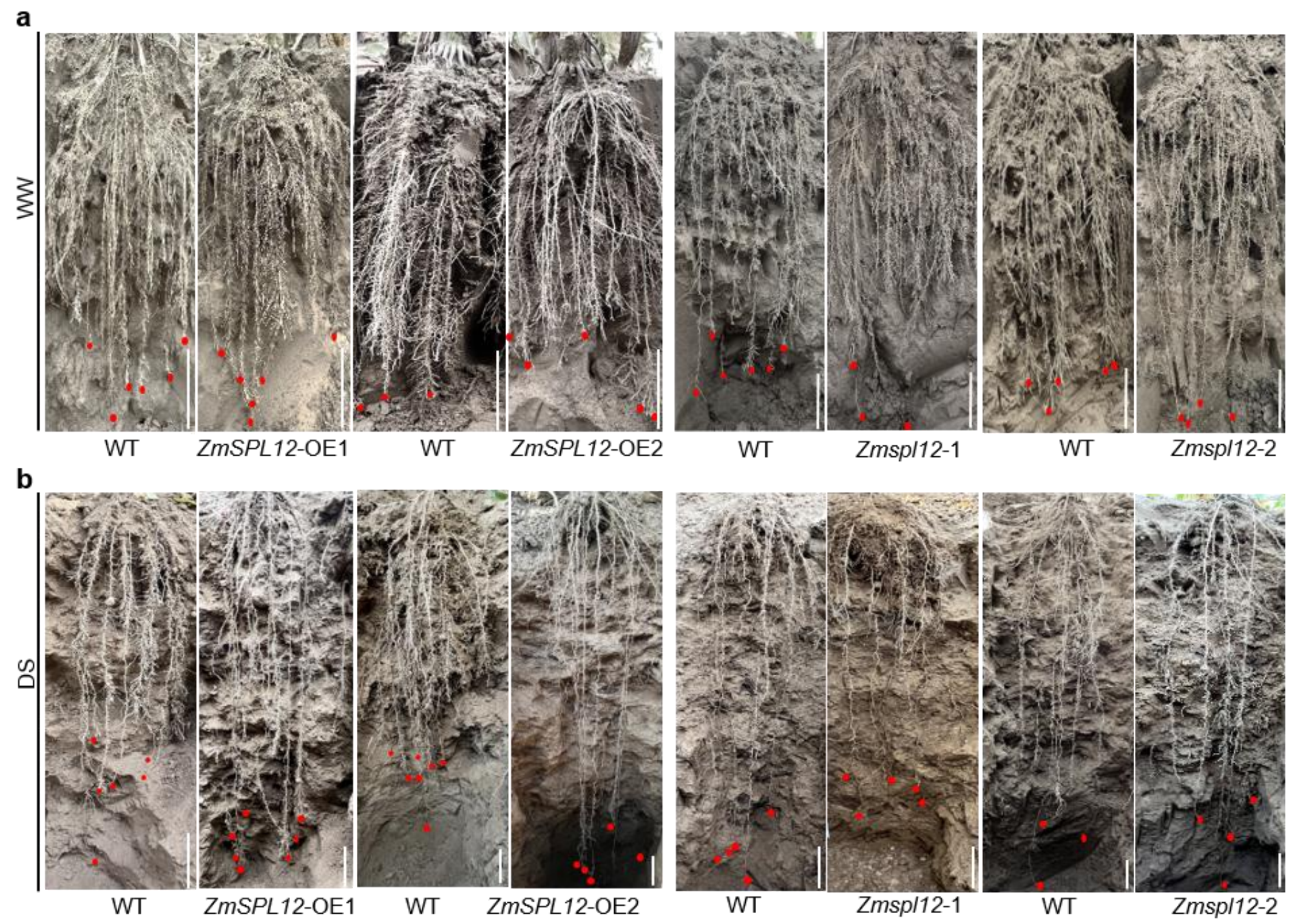

Figure S3. Photographs of crown roots under WW and DS conditions.

a. Representative photographs of *ZmSPL12*-OE (*ZmSPL12*-OE1, *ZmSPL12*-OE2) lines, their wild types, the *ZmSPL12*-KO (*Zmspl12*-1, *Zmspl12*-2) lines and their wild types of crown roots in fields under WW conditions. Scale bars, 10 cm. **b**. Representative photographs of *ZmSPL12*-OE (*ZmSPL12*-OE1, *ZmSPL12*-OE2) lines, their wild types, the *ZmSPL12*-KO (*Zmspl12*-1, *Zmspl12*-2) lines and their wild types of crown roots in fields under DS conditions. Scale bars, 10 cm.

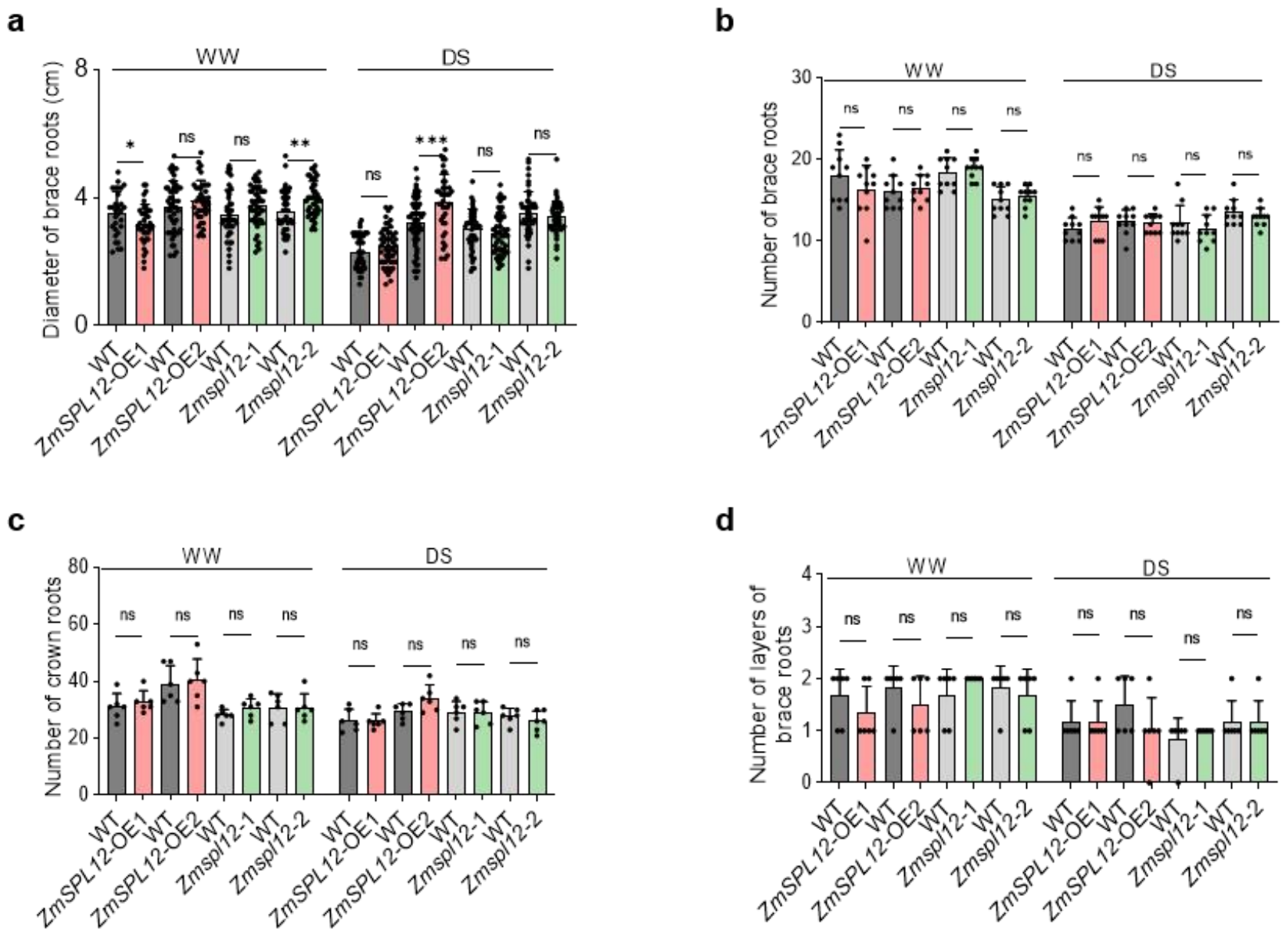

Figure S4. Brace and crown root architectural traits under well-watered and severe drought stress conditions in the field.

a. Diameter of brace roots from plants grown under WW or severe DS conditions in the field (n = 10 plants). **b**. Number of brace roots from plants grown under WW or severe DS conditions in the field (n = 10 plants). **c**. Number of crown roots from plants grown under WW or severe DS conditions in the field (n = 6 plants). **d**. Number of layers of brace roots from plants grown under WW or severe DS conditions in the field (n = 6 plants). In **a**–**d**, values are means ± s.d., with individual data points shown as black circles. Statistical significance of differences was determined by two-tailed Student's t-test. The asterisk indicates significant differences compared to the wild type: *$P<0.05$, **$P<0.01$, ***$P<0.001$.

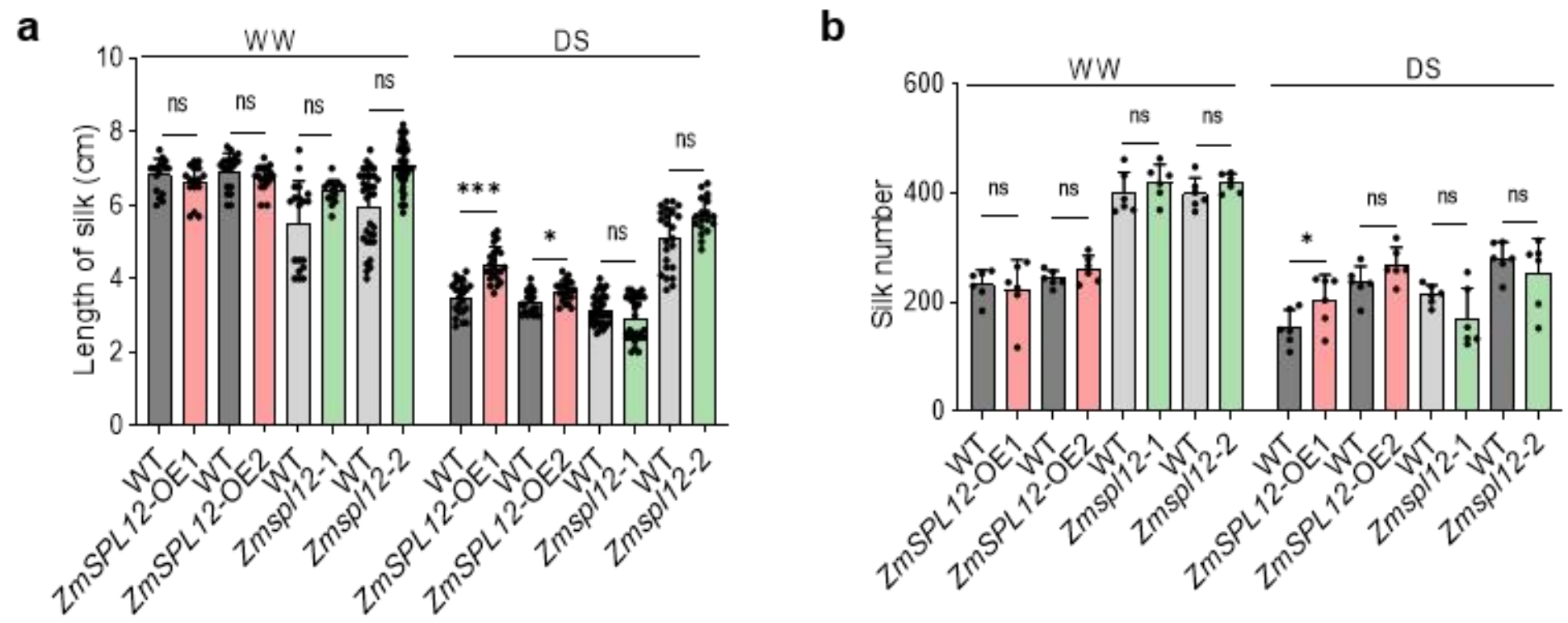

Figure S5. Number and length of silks emerging from *ZmSPL12*-OE lines under drought stress.

a. Length of silk emerged from plants grown under WW or severe DS conditions in the field (n = 6 plants). **b**. Number of silks from plants grown under WW or severe DS conditions in the field (n = 6 plants). In **a**–**b**, values are means ± s.d., with individual data points shown as black circles. Statistical significance of differences was determined by two-tailed Student's t-test. The asterisk indicates significant differences compared to the wild type: *P<0.05, **P<0.01, ***P<0.001.

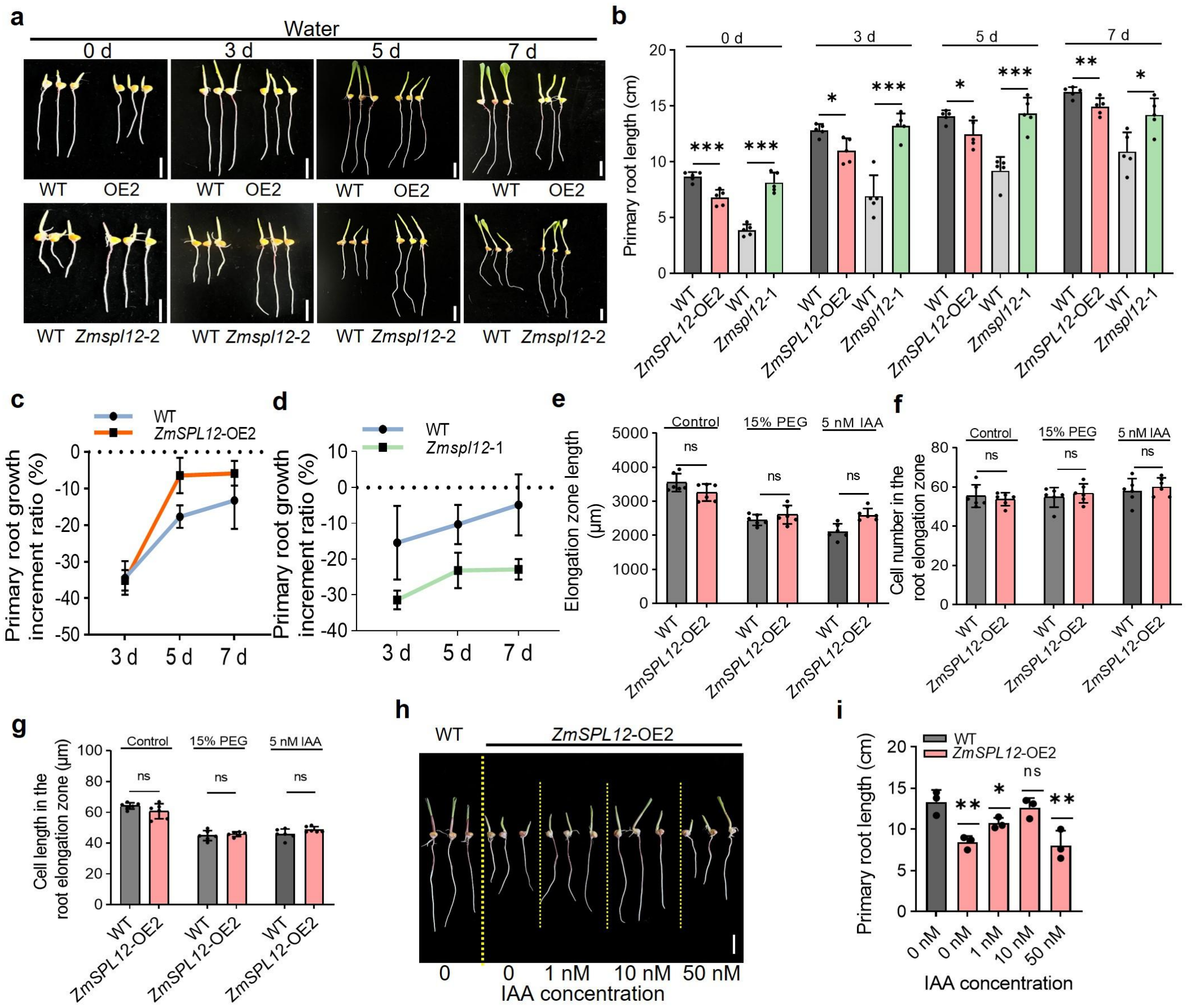

Figure S6. Overexpression of *ZmSPL12* decreased auxin content.

a. Representative photos of 5-day-old *ZmSPL12*-OE2 and its wild type (top), *Zmspl12*-2 and its wild type (bottom) under normal condition (water only). Scale bars, 3 cm. **b**. Primary root length of *ZmSPL12*-OE2 and its wild type, *Zmspl12*-2 and its wild type under normal condition. **c**. Primary root growth increment ratio of *ZmSPL12*-OE2 and its wild type under different treatment times with 15% PEG6000 (*n* =6 plants). **d**. Primary root growth increment ratio of *Zmspl12*-2 and its wild type under different treatment times with 15% PEG6000 (*n* =6 plants). **e**. Lengths of the elongation regions in the root tips of *ZmSPL12*-OE2 and its wild type seedlings under normal conditions, 15% PEG6000 treatment, 3-day 5 nM IAA treatment (*n*=3 biological replicates). **f**. Cell numbers in the elongation regions of root tips from *ZmSPL12*-OE2 and its wild type under normal conditions, 15% PEG6000 treatment, 3-day 5 nM IAA treatment (*n* = 3 biological replicates). **g**. Cell length in the elongation regions of root tips from *ZmSPL12*-OE2 and its wild type under normal conditions, 15% PEG6000 treatment, 3-day 5 nM IAA treatment (*n*=3 biological replicates). **h**. Representative photographs of the primary roots of *ZmSPL12*-OE and its wild type under different concentrations of IAA treatment. Scale bars, 3 cm. **i**. Primary root length of *ZmSPL12*-OE and its wild type under different concentrations of IAA treatment. In **b**, **e**, **f**, **g**, **i**, values are means ± s.d. with individual data points shown as black circles. Statistical analysis was performed using two-tailed Student's *t*-test. The asterisk indicates significant differences compared to the wild type: *P<0.05, **P<0.01, ***P<0.001.

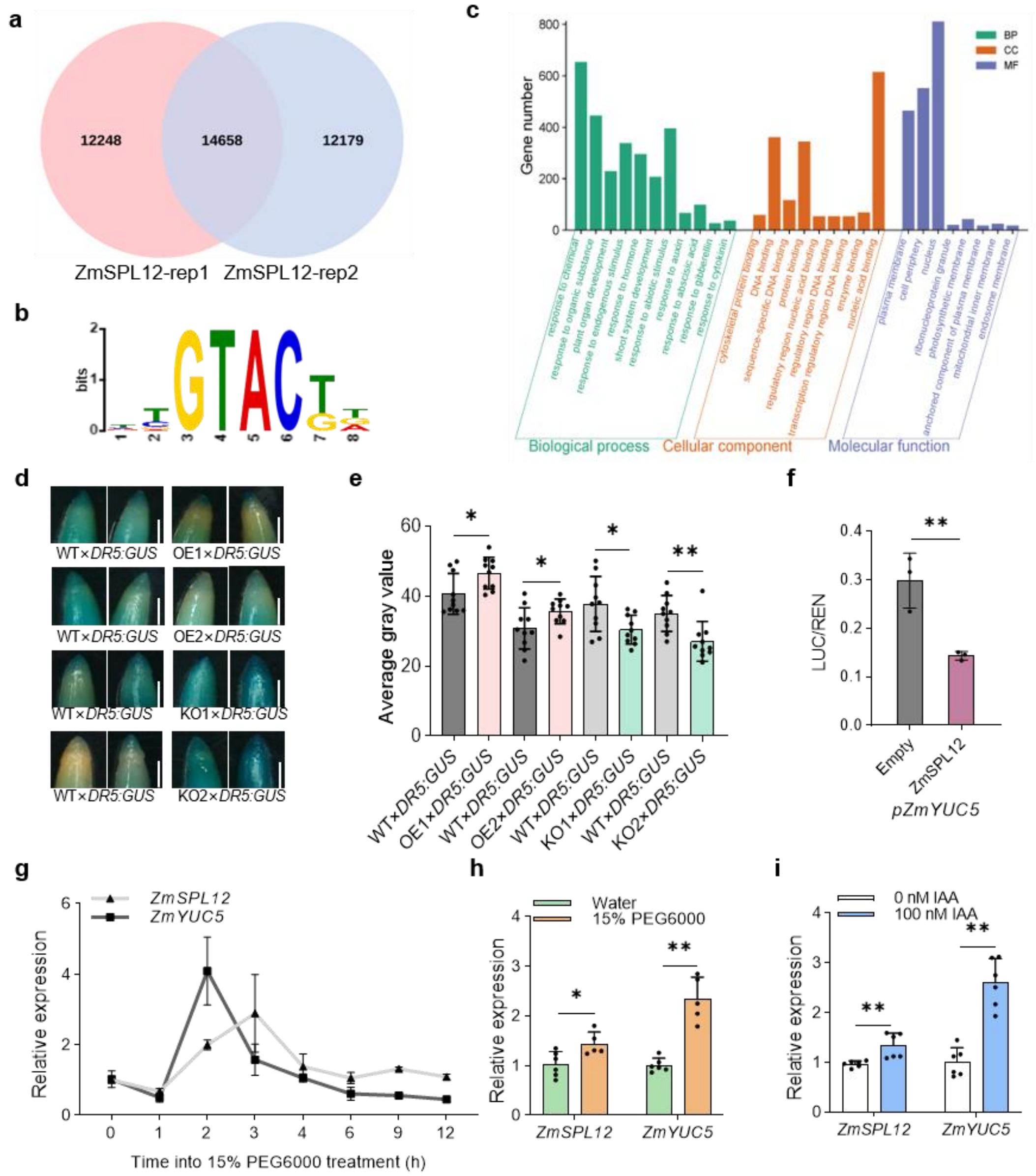

Figure S7. Identification of potential ZmSPL12 direct targets.

a. Venn diagram showing the extent of overlap in ZmSPL12-occupied peaks using two biological replicates of DAP-seq. **b**. Putative DNA-binding motif of ZmSPL12, identified by MEME-ChIP, using the promoter sequences of ZmSPL12-bound genes. **c**. Gene Ontology (GO) term enrichment analysis for genes with promoters that are bound by ZmSPL12. BP, biological process; CC, cellular component; MF, molecular function. **d**. GUS staining of different hybrids between the inbred line harboring the *DR5:GUS* reporter and the indicated lines. Scar bars, 1 cm. **e**. Quantitative of GUS staining of DR5 hybrid (n = 10 biological replicates). **f**. Dual-luciferase reporter assays in maize protoplasts showing that ZmSPL12 inhibits *ZmYUC5* transcription (n = 3 biological replicates), LUC/REN values. **g**. Relative expression levels of *ZmSPL12* and *ZmYUC5* under 15% PEG6000 treatment in root tip of wild type (n = 3 biological replicates). **h**. Relative expression levels of *ZmSPL12* and *ZmYUC5* under normal and 15% PEG6000 treatment in root tip (n =3 biological replicates). **i**. Relative

expression levels of *ZmSPL12* and *ZmYUC5* under different IAA concentrations treatments (n =3 biological replicates). In **e-f**, values are means ± s.d., with individual data points shown as black circles. Statistical significance of differences was determined by two-tailed Student's *t*-test. The asterisk indicates significant differences compared to the wild type: *$P<0.05$, **$P<0.01$.

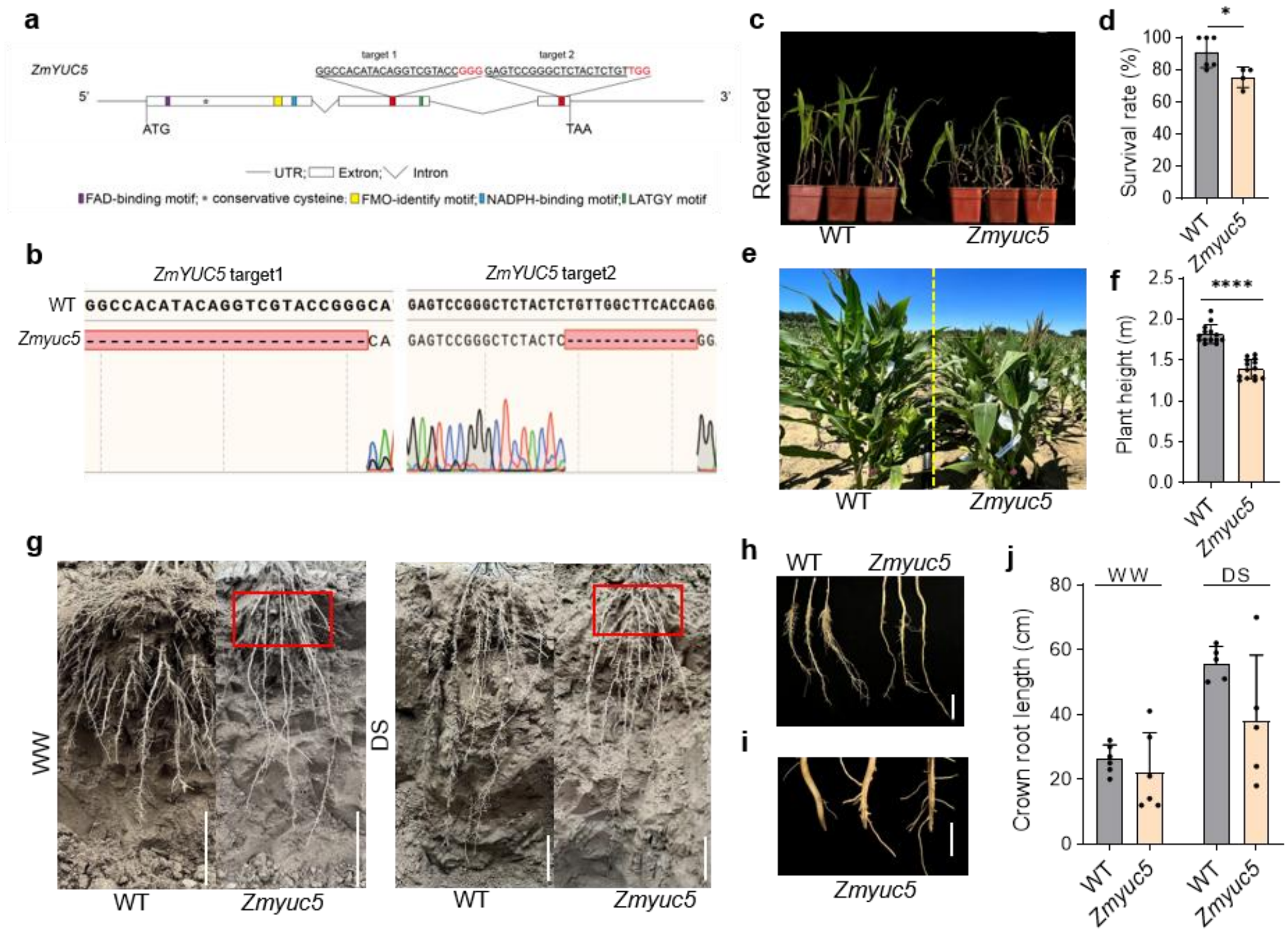

Figure S8. Plant height and crown root performance in the field of *Zmyuc5* and its wild type.

a. Diagram showing the functional motif and sgRNA target sites on the *ZmYUC5* gene. **b**. Sequence analysis of the target sites for homozygous *ZmYUC*5 knockout mutant. **c**. Representative photographs of the *ZmYUC5* knockout mutant line and its wild type (*Zmyuc5,* WT) after rewatering for 3 days. Scale bars, 3 cm. **d**. Survival rates of the *ZmYUC5* knockout mutant line and its wild type after rewatered 3 days. **e**. Plant performance in the field. **f**. Plant height of *ZmYUC5* knockout mutant line and its wild type. **g**. Representative photographs of *ZmYUC5* knockout mutant line and its wild type in fields under WW (left) and DS (right) conditions, the red box represents the roots with damaged tips in *Zmyuc5.* Scale bars, 10 cm. **h**. Representative photographs of roots of the *ZmYUC5* knockout mutant line and its wild type. Scale bars, 3 cm. **i**. Root tip performance of *ZmYUC5* knockout mutant line in the field. Scale bars, 1 cm. **j**. Crown root length of the *ZmYUC5* knockout mutant line and its wild type under WW and DS conditions. In **d**, **f**, **j**, values are means ± s.d. with individual data points shown as black circles. Statistical analysis was performed using two-tailed Student's *t*-test. The asterisk indicates significant differences compared to the wild type: *P<0.05, **P<0.01, ***P<0.001, ****P<0.0001.

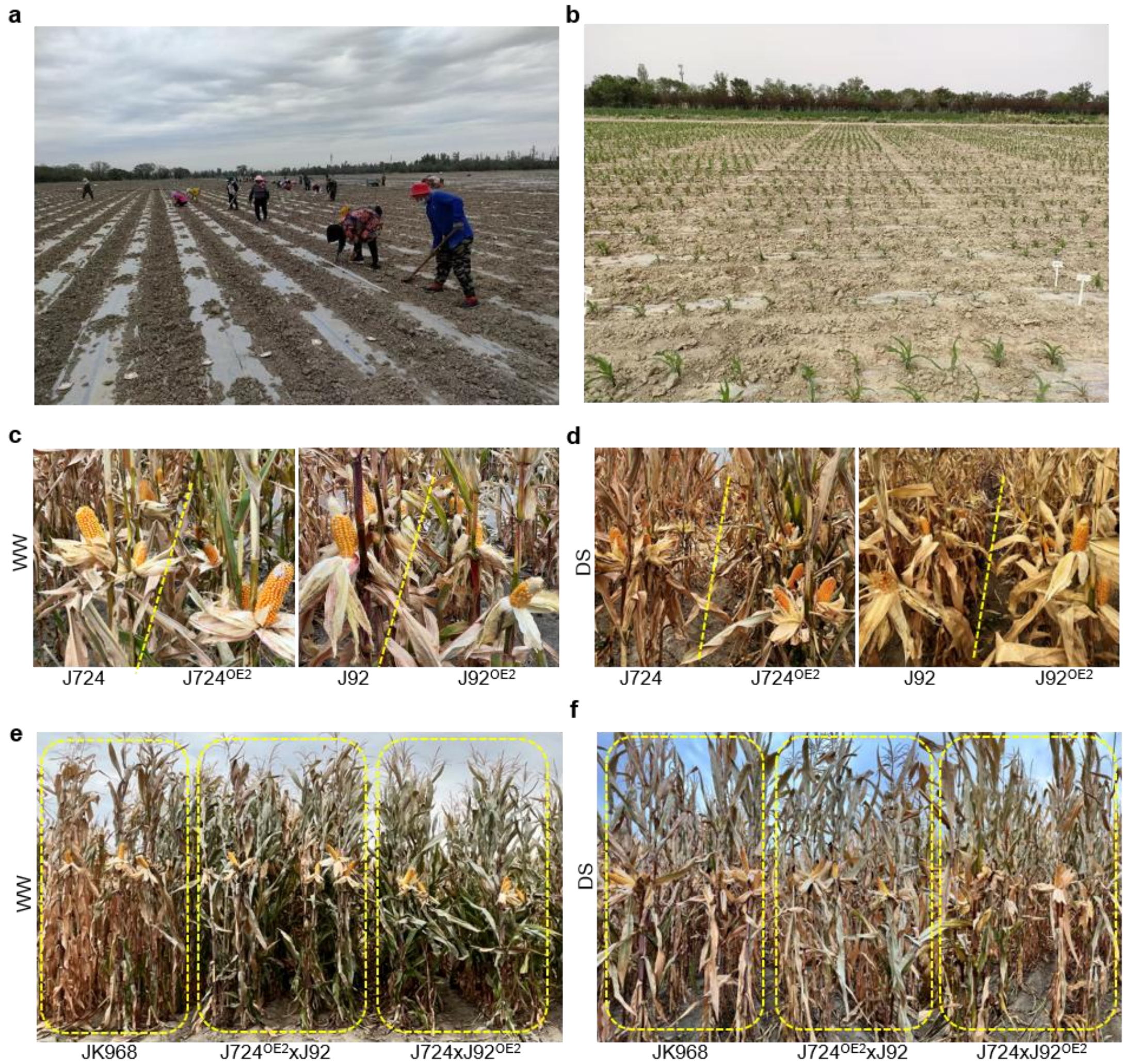

Figure S9. Field sowing and emergence situation in Urumqi, Xinjiang.

a. Sowing in the field. **b**. The emergence situation of seedlings in the field. **c**. Photographs of elite inbred lines ears harvested from plants growing under WW condition. **d**. Photographs of elite inbred lines ears harvested from plants growing under DS condition. **e**. Photographs of hybrids ears harvested from plants growing under WW condition. **f**. Photographs of hybrids ears harvested from plants growing under DS condition.

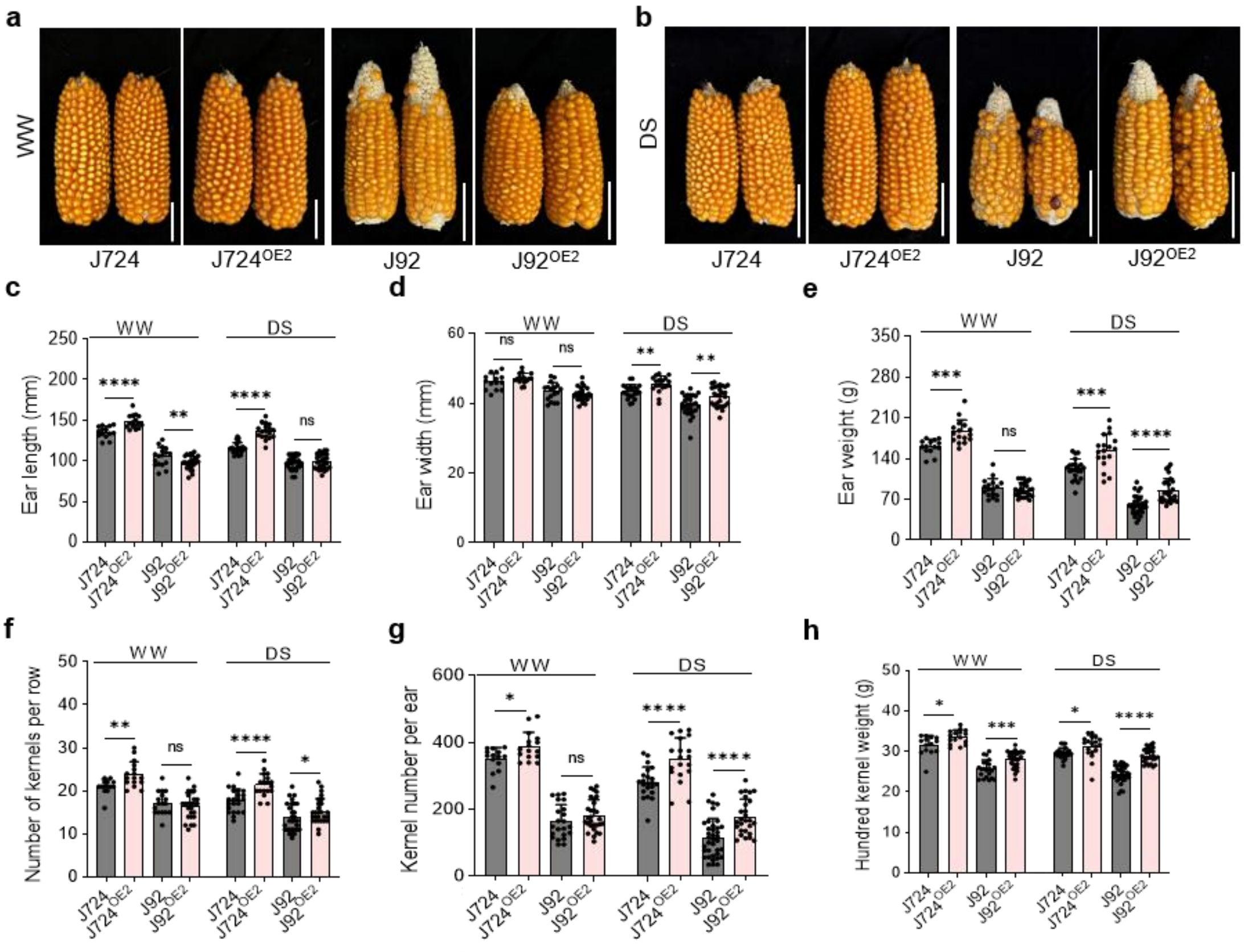

Figure S10. The improved elite parental inbred lines carrying the *ZmSPL12*-OE transgene show better drought resistance than the parental lines.

a, **b**. Representative photographs of improved inbred lines under WW (**a**) or moderate DS (**b**) conditions. Scale bars, 3 cm. **c**–**h**. ear length (**c**), ear width (**d**), ear weight (**e**), number of kernels per row (**f**), kernel number per ear (**g**) and hundred-kernel weight (**h**) of the *ZmSPL12* improved inbred lines and their respective controls under WW and moderate DS conditions in the field ($n \geq 15$ plants). In **c**-**h**, values are means ± s.d., with individual data points shown as black circles. Statistical significance of differences was determined by two-tailed Student's *t*-test. The asterisk indicates significant differences compared to the wild type: *P<0.05, **P<0.01, ***P<0.001, ****P<0.0001.

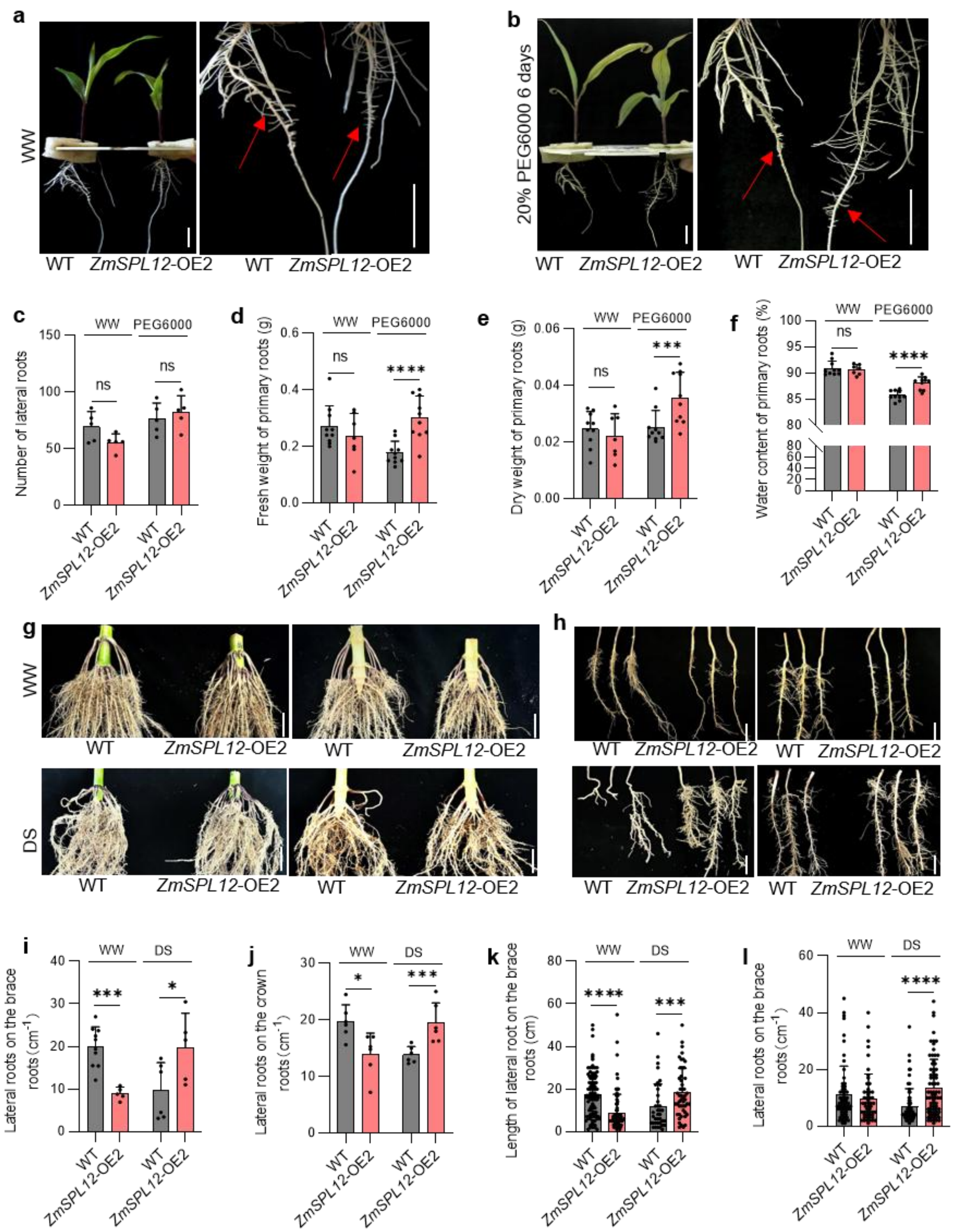

Figure S11. *ZmSPL12* positively regulates lateral root number and length of maize under drought stress.

a. Representative photographs of seedlings from the *ZmSPL12*-OE2 and its wild type under well-watered (WW) conditions. Scale bars, 3cm. **b**. Representative photographs of seedlings from the *ZmSPL12*-OE2 and its wild treated with 20% PEG6000 for 6 days. Scale bars, 3cm. **c**-**f**. Number of lateral roots (**c**), Dry weight of primary roots

(**d**), Fresh weight of primary roots (**e**), Water content of primary roots (**f**). **g**. Representative photographs of *ZmSPL12*-OE2 its wild type plants of the profile of roots under WW condition (top) and DS condition (bottom). Scale bars, 3cm. **h**. Representative photographs of *ZmSPL12*-OE2 and its wild type of lateral root on the brace roots (left) and crown roots (right) under WW condition (top) and DS condition (bottom). Scale bars, 3cm. **i-l**. Lateral root density on the brace roots (**i**), Lateral root density on the crown roots (**j**), Lateral root length on the brace roots (**k**), Lateral root length on the crown roots (**l**). In **c**–**f**, **i-l**, values are means ± s.d., with individual data points shown as black circles. Statistical significance of differences was determined by two-tailed Student's *t*-test. The asterisk indicates significant differences compared to the wild type: *P<0.05, **P<0.01, ***P<0.001, ****P<0.0001.

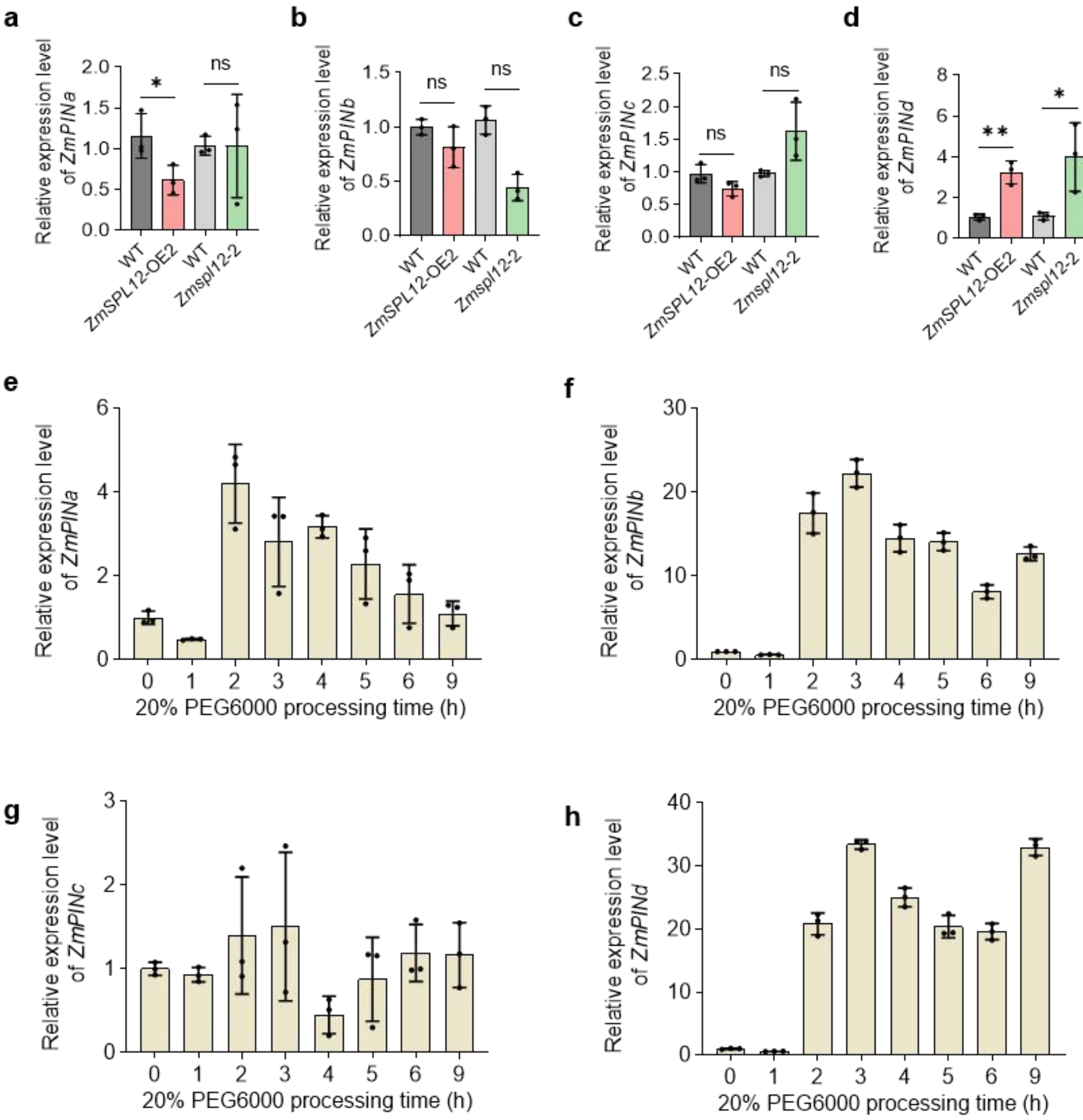

Figure S12. Differential expression of *ZmPINs* in Zm*SPL12*-OE2/*Zmspl12*-2 under normal conditions and in wild type under osmotic stress.

a-**d**. Relative expression level of *ZmPINa* in primary root of *ZmSPL12*-OE2, *Zmspl12*-2, and their wild types under normal conditions (*n* = 3 biological replicates) (**a**); Relative expression level of *ZmPINb* in primary root of *ZmSPL12*-OE2, *Zmspl12*-2, and their wild types under normal conditions (*n* = 3 biological replicates) (**b**); Relative expression level of *ZmPINc* in primary root of *ZmSPL12*-OE2, *Zmspl12*-2, and their wild types under normal conditions (*n* = 3 biological replicates) (**c**); Relative expression level of *ZmPINd* in primary root of *ZmSPL12*-OE2, *Zmspl12*-2, and their wild types under normal conditions (*n* = 3 biological replicates) (**d**). **e**-**h**. Relative expression level of *ZmPINa* in primary root of the wild type under 20% PEG6000 treatment for the indicated times (*n* = 3 biological replicates) (**e**); Relative expression level of *ZmPINb* in primary root of the wild type under 20% PEG6000 treatment for the indicated times (*n* = 3 biological replicates) (**f**); Relative expression level of *ZmPINc* in primary root of the wild types under 20% PEG6000 treatment for the indicated times (*n* = 3 biological replicates) (**g**); Relative expression level of *ZmPINd* in primary root of the wild types under 20% PEG6000 treatment for the indicated

times (n = 3 biological replicates) (**h**). In **a**–**d**, values are means ± s.d., with individual data points shown as black circles. Statistical significance of differences was determined by two-tailed Student's *t*-test. .The asterisk indicates significant differences compared to the wild type: *P<0.05, **P<0.01.
